# Fecal microbiota transplantation and *Akkermansia muciniphila* restore neurodevelopment and behavior *via* the gut-brain axis in autism-like zebrafish

**DOI:** 10.1093/ismejo/wrag074

**Published:** 2026-03-30

**Authors:** Pan-Pan Jia, Yan Li, Hao-Yu Yang, Yuan Ding, Feng-Yi Guo, Ming-Fei Wu, Jin-Qiu Jia, De-Sheng Pei

**Affiliations:** School of Public Health, Chongqing Medical University, Chongqing, 400016, China; School of Public Health, Chongqing Medical University, Chongqing, 400016, China; School of Public Health, Chongqing Medical University, Chongqing, 400016, China; School of Public Health, Chongqing Medical University, Chongqing, 400016, China; School of Public Health, Chongqing Medical University, Chongqing, 400016, China; School of Public Health, Chongqing Medical University, Chongqing, 400016, China; School of Public Health, Chongqing Medical University, Chongqing, 400016, China; School of Public Health, Chongqing Medical University, Chongqing, 400016, China; Chongqing Miankai Biotechnology Research Institute Co., Ltd., Chongqing, 400025, China

**Keywords:** *Akkermansia muciniphila*, Autism spectrum disorder, Fecal microbiota transplantation, Germ-free zebrafish, Microbiota-gut-brain axis

## Abstract

Effective therapies for Autism Spectrum Disorder (ASD) are currently limited, and the functional connections between gut microbiota and brain development are not fully elucidated. Using the Katnal2 mutant zebrafish as an ASD-like model, we evaluated whether fecal microbiota transplantation (FMT) from wild-type donors or supplementation with the probiotic *Akkermansia muciniphila* (*A. muciniphila*) could ameliorate neurodevelopmental deficits. Assessments included developmental phenotypes, behavior, microbial profiling, neurotransmitter-related gene expression, and short-chain fatty acid (SCFA) signaling in conventionally reared (CR) and germ-free (GF) fish. FMT from wild-type donors and *A. muciniphila* supplementation significantly improved hatching rates, growth parameters, heart rate, and locomotor activity in Katnal2 mutants, whereas microbiota from Katnal2 mutants induced analogous deficits in wild-type recipients. *A. muciniphila* successfully colonized the gut, reshaped microbial communities, and reduced anxiety-like behaviors. Mechanistically, *A. muciniphila* upregulates genes involved in dopamine (*th*), serotonin (*tph1a*), and gamma-aminobutyric acid (GABA) synthesis, downregulates the serotonin receptor *htr3a*, and enhances expression of the SCFA receptor *ffar2*, independently of total SCFA levels. Correlation analyses linked key developmental, behavioral, and transcriptional changes to altered microbial genera in a sample-specific manner, highlighting compositionally driven neuromodulatory effects of genetic and probiotic interventions. Thus, microbiota-targeted intervention with *A. muciniphila* rescues neurodevelopmental impairments in ASD models by remodeling the gut-brain axis, supporting its translational potential.

## Graphical Abstract

**Graphical Abstract:**
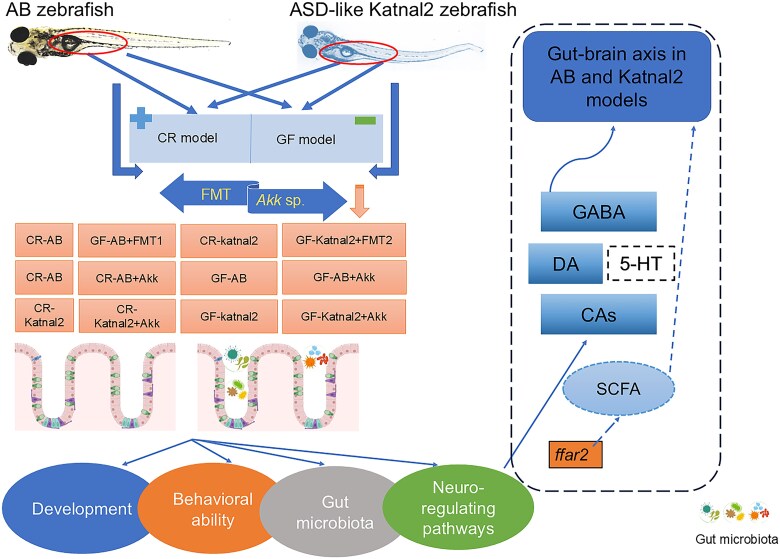

## Introduction

Autism spectrum disorder (ASD) is a complex neurodevelopmental condition defined by impairments in social communication and interaction, alongside restricted and repetitive behaviors [1–3]. Affecting 1%–2% of children worldwide, ASD is frequently accompanied by intellectual disability, epilepsy, anxiety, and sleep disturbances [4–6]. Despite extensive genetic and clinical research, its etiology remains poorly understood, and effective therapies are lacking. Both heritable and environmental factors contribute to ASD, with de novo mutations, maternal immune activation, and prenatal exposures implicated in altering early brain development [7, 8]. This multifactorial origin underscores the urgent need to identify modifiable pathways that influence neurodevelopment and behavior.

Among the many risk genes, *Katnal2* has emerged as a strong candidate for ASD susceptibility, playing key roles in neurodevelopment [9]. The human KATNAL2 is a risk gene for ASD, which encodes a microtubule-severing protein essential for cell division, ciliary function, and neural development [10, 11]. Knockout of *Katnal2* in multiple animal models, such as *Xenopus, mice,* and zebrafish (*Danio rerio*), was applied in autism-related phenotypes research, which exhibited developmental abnormalities, reduced brain size, damaged nervous system, and impaired locomotor activity [11–13]. Zebrafish, owing to their genetic conservation with humans and suitability for high-throughput behavioral and microbiota studies [14–16], provide a powerful in vivo model for dissecting the consequences of *Katnal2* mutations [17, 18]. The Katnal2 zebrafish mutant has been systematically validated and exhibits observable behavioral phenotypes, providing a valuable genetic tool for modeling human disorders, identifying potential biomarkers, and probing the effects of probiotic interventions [13].

In parallel with genetic risk factors, accumulating evidence implicates the gut microbiota as a key regulator of neurodevelopment *via* the microbiota-gut-brain axis [19, 20]. The gut microbiota and central nervous system develop in parallel during early life, a critical period that coincides with the onset of ASD symptoms [21]. Perturbations in microbial composition can disrupt immune, endocrine, and neural signaling, resulting in altered neurotransmitter metabolism and behavioral abnormalities [22]. The relative abundance of microbiota in patients with ASD was different from that of normal individuals, whose amount was related to the severity of ASD’s restrictive/repetitive behavior [23]. Fecal microbiota transplantation (FMT) from ASD patients into germ-free (GF) zebrafish and mice induced autism-like behaviors, while probiotic supplementation was reported to improve communication and social interaction in children with ASD [24, 25]. The wild type (AB strain) and Katnal2 lines, combined with conventionally reared (CR) and GF models, zebrafish larvae that are beneficial for observing microbial colonization, can provide an in-depth understanding of microbiota-host interactions. Thus, we suspected that positive changes of intestinal microbiota disorders, such as FMT from the healthy donors and probiotics, possibly contribute to intestinal permeability, immunity enhancement, and autism symptoms in ASD and microbial deficit models, which may represent a promising therapeutic avenue.

In the central nervous system, gamma-aminobutyric acid (GABA), dopamine (DA), and serotonin (5-HT) pathways potentially regulate stress responses, anxiety, and aggressive behavior [18, 26]. Among the related genes, *tph1a* is involved in the synthesis of 5-HT in both the peripheral and central nervous systems [27, 28], whose altered expression may lead to metabolic disorders and then elevated peripheral levels. Compared to normal children, autistic children exhibit significantly higher *tph1a* expression and lower *htr3a* levels [29]. In neuro-regulation, the DA pathway involves multiple physiological functions, such as motor control, cognitive functions, and reproductive behaviors. The DA (a neurotransmitter, synthesized in both central and peripheral nervous systems, and exerts effects through binding to G protein-coupled receptors) signaling pathway is crucial for maintaining physiological processes; imbalances may lead to functional impairments associated with neurological disorders. Tyrosine is converted into DA *via* tyrosine hydroxylase (*th*). This study further investigated whether the impact of FMT between AB and Katnal2 zebrafish involves these neuro-regulation pathways, including brain development-related *pah*, *dhfr*, *htr3a*, and tph1a, *th* (DA pathway), *gad1b* (GABA pathway), and carbonic anhydrase (*ca2*, *ca4a*, *ca7*, *ca14*) genes.

*Akkermansia muciniphila*, a mucin-degrading bacterium first isolated in 2004, has been recognized as a “next-generation probiotic” [30]. Abundant in the intestinal mucosa, *A. muciniphila* contributes to barrier integrity, immune regulation, and metabolic homeostasis [31]. Altered abundance of this bacterium has been linked to metabolic and neurological disorders, and supplementation has shown potential to restore microbial balance and alleviate behavioral symptoms *via* the gut-brain axis [32, 33]. In ASD, the altered gut microbial profile and the key metabolites were associated with abnormal neurotransmitter metabolism, including dopamine, histidine, and GABA [34]. Microbial metabolites, particularly short-chain fatty acids (SCFAs), mediate gut-brain communication by activating specific receptors, including Free Fatty Acid Receptor 2 (FFAR2), which in turn modulate key nervous system signaling pathways [35, 36]. Whether *A. muciniphila* can directly modulate neurodevelopmental outcomes in ASD-like animal and GF models remains unknown.

Here, we investigated the therapeutic potential of fecal microbiota transplantation (FMT) and *A. muciniphila* supplementation in *Katnal2* mutant zebrafish, a validated ASD-like model. Using CR and GF zebrafish, we performed reciprocal FMT between wild-type AB and *Katnal2* lines, tracked *A. muciniphila* colonization, and systematically assessed developmental indices, locomotor activity, microbial composition, neurotransmitter-related gene expression, and SCFA signaling. Our findings demonstrated that microbial interventions can reprogram the microbiota-gut-brain axis to rescue neurodevelopmental and behavioral deficits, highlighting the precise regulation by FMT and probiotics like *A. muciniphila* as promising microbial therapeutic candidates for related autism defects.

## Materials and methods

### Ethics statement

All zebrafish experiments were approved by the Institutional Animal Care and Use Committee of Chongqing Medical University and performed in accordance with relevant guidelines to ensure animal welfare.

### Zebrafish maintenance and GF models

Katnal2^−/−^ zebrafish were obtained from Prof. Xiong Bo (Huazhong University of Science and Technology) and maintained at 28°C under a 14:10 light/dark cycle. Adult males and females were paired overnight at a 1:2 ratio in spawning tanks, and fertilized eggs were collected the following morning following light stimulation. Embryos were incubated at 28°C with daily water changes and removal of dead embryos; larvae were fed paramecia at 3 dpf and *Artemia* at 10 dpf (hatched under standard saline, aerated conditions at 28 ± 0.5°C for 48 h, with eggshells removed before feeding). GF zebrafish were generated from healthy embryos using our optimized sterile protocol (detailed in Text S1) [37]. Sterility was confirmed by culturing samples in TSB and BHI broth under oxic and anoxic conditions at 28°C for ≥72 h, and by plating on TSA and blood agar plates for ≥120 h. PCR assays were routinely performed to confirm the absence of bacterial contamination, using bacterial samples as positive controls.

### FMT between AB and Katnal2 zebrafish lines

Fecal matter for transplantation was collected from adult zebrafish after overnight housing in spawning tanks. The collection and processing protocol was adapted from established FMT methods [25, 38] before administration to GF recipients. Briefly, fresh fecal samples (100 mg) collected from adult AB and Katnal2 zebrafish were suspended in 10 ml phosphate-buffered saline (PBS), vortexed, and centrifuged at 500 g for 5 min. Then, the fresh supernatant was stored at 4°C for a moment before use, and long-term stored within 50% glycerol at −80°C. Fertilized eggs of AB and Katnal2 zebrafish were collected and divided into four groups (n = 100 each): AB control (CR-AB), Katnal2 control (CR-Katnal2), germ-free AB transplanted with Katnal2 feces (GF-AB+FMT1), and germ-free Katnal2 transplanted with AB feces (GF-Katnal2+FMT2). Control groups of CR-AB and CR-Katnal2 were maintained under standard conditions, while GF groups were generated according to an optimized sterile protocol (detailed in Text S1). At 5 dpf, GF-AB and GF-Katnal2 larvae had reached the mouth-opening stage with mature intestinal function, suitable for FMT and bacterial colonization studies [25, 39]. Larvae in the GF-AB and GF-Katnal2 groups, housed in 6-well plates, received 200 μL per well of the respective fecal supernatant, which was added to the culture medium during daily renewal. At 7 dpf, zebrafish from all experimental groups were sampled, flash-frozen in liquid nitrogen, and stored at −80°C for subsequent analysis. These samples were used for 16S rRNA genes sequencing of microbial composition, quantification of SCFAs, and RT-PCR analysis of gene expression.

### Cultivation, identification, gram staining, and exponential growth of *A. muciniphila*

*After activation,*  ***A. muciniphila***  *was cultured anoxically in mucin-supplemented BHI broth (37°C, 24–72 h), and growth was monitored by OD600 to establish exponential phase kinetics. Colonies were Gram-stained for morphological assessment, and genomic DNA was extracted for 16S rRNA genes amplification (27F/1492R), sequencing, and phylogenetic confirmation against the NCBI database. Bacterial concentration was determined by correlating OD600 with colony-forming units: cultures in exponential phase were serially diluted (10^−1^–10^−7^), plated on mucin-BHI agar, and incubated anoxically at 37°C, with CFU counts averaged across triplicate plates in three independent experiments.* The bacterial concentration corresponding to OD600 absorbance was calculated using the following formula:


$$ \mathrm{C}=\left(\mathrm{N}\div \mathrm{V}\right)\times \mathrm{M} $$

where C is the bacterial solution concentration, N is the average number of colonies on the plate at a given dilution, V is the volume of dilution used for plating (mL), and M is the dilution factor.

### Fluorescence labeling and *in vivo* colonization of *A. muciniphila* in AB and Katnal2 zebrafish

Fresh bacterial suspensions were labeled with CM-DiI dye (10 μg/mL in PBS, 0.5% DMSO) for 20 min at 37°C, followed by 15 min at 4°C, washed by repeated centrifugation (12 000 rpm, 1 min), and examined under a fluorescence stereomicroscope to confirm labeling. At 5 dpf, 20 larvae per well were transferred to 6-well plates, and fluorescence imaging was performed every 24 h to track intestinal colonization of *A. muciniphila* for 72 h. Retention and localization within the gut were quantified by fluorescence intensity.

### Developmental indices and movement ability of FMT and *A. muciniphila*-treated fish

The growth and developmental indices of zebrafish were recorded daily, including survival rate, hatching rate, body length, body weight, and heartbeats per 10 s. At 7 dpf, six larvae were randomly selected from each group for the FMT experiment (CR-AB, CR-Katnal2, GF-AB+FMT1, GF-Katnal2 + FMT2) and the *A. muciniphila* treatment (CR-AB, CR-AB+Akk, GF-AB, GF-AB+Akk), and placed individually in 24-well plates (one larva per well). After 30 min of acclimatization, measurements were taken simultaneously across all groups. Zebrafish movements were recorded using high-speed infrared cameras operating at 157 frames per second under two lighting conditions: complete darkness for 10 min and alternating light–dark cycles for 50 min. Video data were analyzed using EthoVision XT tracking software (Noldus, Wageningen, Netherlands).

### Composition of microbiota in AB and Katnal2 zebrafish before and after *A. muciniphila* treatment

The total genomic DNA was extracted from zebrafish using the E.Z.N.A. Soil DNA Kit. The concentration and purity of the extracted DNA were verified by 1% agarose gel electrophoresis. The V3-V4 hypervariable region of the 16S rRNA gene was amplified using barcoded primers 341F (5′-CCTAYGGGRBGCASCAG-3′) and 806R (5′-GGACTACNNGGGTATCTAAT-3′). All PCR reactions were carried out using Phusion High-Fidelity PCR Master Mix (New England Biolabs) under the following thermal cycling conditions: initial denaturation at 98°C for 1 min; 30 cycles of denaturation at 98°C for 10 s, annealing at 50°C for 30 s, and elongation at 72°C for 30 s; and a final extension at 72°C for 5 min. PCR products were detected by electrophoresis on a 2% agarose gel, then pooled and purified to construct sequencing libraries using the NEB Next Ultra DNA Library Prep Kit (Illumina, USA). Library quality was assessed on an Agilent 5400 system (Agilent Technologies, USA), and sequencing was performed on an Illumina platform to generate 250 bp paired-end reads. Bioinformatics analysis was performed by merging and processing raw FASTQ files using the QIIME 2 *dada2* plugin to generate a feature table of amplicon sequence variants (ASVs). Differential abundance analysis was conducted using ANOVA, LEfSe, and DESeq2 to identify bacterial taxa with significant differences among groups. Alpha diversity indices were calculated at the feature level to estimate microbial diversity within individual samples, while principal coordinate analysis (PCoA) was used to visualize structural variation in microbial communities across samples. Spearman correlation analysis was employed to assess associations between bacterial taxa and key physiological indices. In addition, potential functional profiles of microbial communities were predicted using PICRUSt based on KEGG Ortholog (KO) annotations (Test S2).

### Expression levels of key genes of neuro-regulation pathways in zebrafish

Total RNA was extracted from zebrafish larvae using Trizol reagent, and purity was confirmed by spectrophotometry (A260/280 = 1.8–2.1; A260/230 > 1). RNA was reverse-transcribed into cDNA using a commercial reverse transcription kit, and RT-PCR quantified gene expression with SYBR Green (TB Green II) and zebrafish-specific primers (Table 1, designed *via* NCBI Primer). Cycling conditions were 95°C for 3 min, followed by 40 cycles of 95°C for 10 s, 60°C for 20 s, and 72°C for 30 s, with dissociation curve analysis to confirm specificity. All reactions were performed in triplicate, and relative expression was calculated using the 2^-ΔΔCT^ method with *gapdh* as the internal control.

**Table 1:** Primers of zebrafish in this study.

**Gene name**	**Access No.**	**Sequence (5 '-3')**	**Product length (bp)**
*gapdh*	NM_001115114.1	F: TGAGGTTAAGGCAGAAGGCG R: CCCTTAATGTGAGCAGAAGCC	168
*spra*	NM_001024430.2	F: TCCATAACGCCGCTTCTCT R: ACAGCGAGCTGATGTTCACG	183
*dhfr*	NM_131775.1	F: AGCAGAGAACTCAAGACAGCC R: AGCGCTCCATCACCTCCTTA	157
*pah*	NM_200551.1	F: GACCCAGTACATTCGCCACA R: GCGAGCCCAATTTCCTGAGA	132
*th*	NM_131149.1	F: TGGATCAGGATCACCCAGGA R: GTAGACCTCCCGCCATGTTC	149
*tph1a*	NM_178306.3	F: CCTCGGAATGACTTTGGAGGA R: TACAAGGTTTACATGGTTTTCCTGG	199
*ca2*	NM_199215.5	F: GGATCTGCAGACGACAAGGG R: GGTTGTCTGCACCAATCTTCA	169
*ca4a*	NM_001114407.2	F: ACACACCATTGATGGAGAGCA R: TGGTGCTGGACACCTCATAGA	140
*ca7*	NM_200813.1	F: TGCTGTGGCTCCGAACATAC R: GTGCTCATCACCGGTTTCCA	159
*ca14*	NM_001328144.1	F: AGTGCTCAGGAAGTGCAACA R: CTCCCAGGCTGGTTGTATCC	181
*htr3a*	XM_009295409.5	F: AAGAAAACAACATGAACACTGCCAT R: CCTTCTTCTCCAGGAACTGGC	171
*ffar2*	NM_001082895.1	F: CGTCGCATTTCCAATCCGAT R: TCACATGGGGATTGAGCTGT	173

### Measurement of the total content of metabolites SCFAs

Thirty zebrafish per sample were weighed, homogenized in PBS (1:9, w/v), and centrifuged at 3000 rpm for 10 min. Supernatants were stored at −80°C until analysis. The total SCFA level in the samples was quantified using the ELISA kit.

### Statistical analysis

All data were analyzed using SPSS (version 21.0) with appropriate statistical tests, including one-way ANOVA, Student’s t-test, or the nonparametric Kruskal-Wallis test. Developmental indexes, behavioral data, and RT-qPCR were analyzed and visualized using Origin software (Version 2024b) and Graphpad Prism10 with ANOVA (Brown-Forsythe test, Bartlett test, Tukey multiple comparisons). All data were presented with the mean ± standard error (SEM) for each group with at least three replicates. The statistical symbols of ^*^, ^**^, and ^***^ were indicated *P* < .05, .01, and .001, representing significant differences between control and treatment groups.

## Results

### Developmental indices after FMT between AB and Katnal2 zebrafish lines

**Figure 1:**
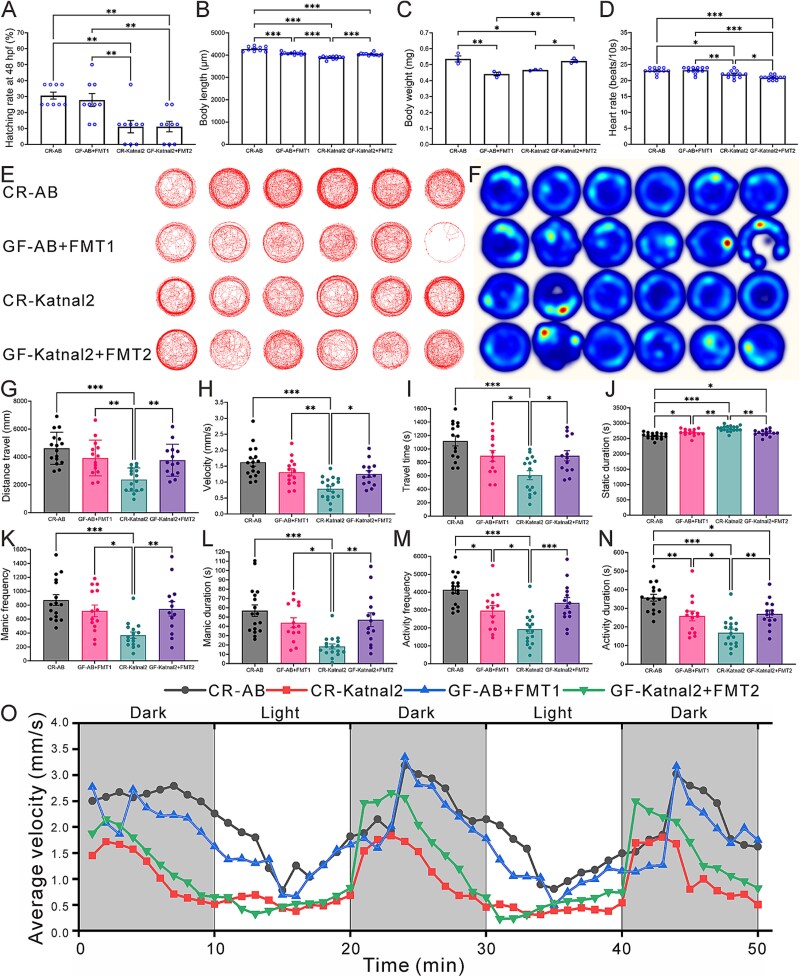
Effects of FMT on development and behavior in AB and Katnal2 zebrafish. (A) Hatching rate at 48 hpf. (B) Body length (μm). (C) Body weight (mg, n = 30 fish/replicate sample). (D) Heart rate (beats/10 s). (E) Trajectory plots. (F) Heat maps. (G) Distance traveled. (H) Velocity. (I) Travel time. (J) Static duration. (K) Manic frequency. (L) Manic duration. (M) Activity frequency. (N) Activity duration. (O) Average velocity (mm/s) in light–dark stimulation test. Data are from 18 zebrafish per group across three independent experiments, with statistical significance assessed at ^*^  *P <* .05, ^**^P < .01, ^***^P < .001 vs. CR-AB, GF-AB+FMT1, CR-Katnal2, and GF-Katnal2 + FMT2.

GF zebrafish were successfully established with no detectable bacterial growth. To evaluate microbiota effects, FMT was performed between AB and Katnal2 fish (Groups: CR-AB as AB control, CR-Katnal2 as Katnal2 control, GF-AB+FMT1 as germ-free AB fish transplanted with Katnal2 feces, and GF-Katnal2 + FMT2 as germ-free Katnal2 fish transplanted with AB feces), and developmental indices were compared with those of the CR controls (Fig. 1). The hatching rate was significantly reduced in CR-Katnal2 (11.1%) *versus* CR-AB (30.5%, *P <* .01), indicating impaired early development (Fig. 1A). Body length was decreased in GF-AB+FMT1 (4084.3 μm), CR-Katnal2 (3890.6 μm), and GF-Katnal2 + FMT2 (4044.0 μm) compared with CR-AB (4278.6 μm, all *P <* .001). However, both FMT groups showed partial rescue relative to CR-Katnal2 (*P <* .001) (Fig. 1B). Body weight showed a similar trend: CR-AB averaged 0.54 mg/30 fish, while GF-AB+FMT1 (0.44 mg, *P <* .01) and CR-Katnal2 (0.47 mg, *P <* .05) were lower; GF-Katnal2 + FMT2 (0.52 mg) exceeded both GF-AB+FMT1 (*P <* .01) and CR-Katnal2 (*P <* .05) (Fig. 1C). Heart rate was reduced in CR-Katnal2 (21.9 beats/10 s, *P <* .05) versus CR-AB (23.1 beats/10 s) and further decreased in GF-Katnal2 + FMT2 (20.9 beats/10 s, *P <* .001). In contrast, GF-AB+FMT1 showed a significant increase, compared to CR-Katnal2 (*P <* .05), while GF-Katnal2 + FMT2 also exhibited partial recovery (*P <* .05) (Fig. 1D).

### Movement ability after FMT between AB and Katnal2 zebrafish

Video tracking revealed distinct locomotor patterns among groups (Fig. 1). GF-AB+FMT1 zebrafish showed sparse, edge-restricted trajectories, compared to CR-AB, while CR-Katnal2 exhibited reduced movement; in contrast, GF-Katnal2 + FMT2 displayed denser trajectories and greater activity than GF-Katnal2 (Fig. 1E and F). Quantitatively, CR-Katnal2 showed significant reductions in distance traveled, velocity, movement duration, manic frequency/duration, and active frequency/duration relative to CR-AB (Fig. 1G–N). Both FMT groups partially rescued these deficits: GF-AB+FMT1 and GF-Katnal2 + FMT2 exhibited longer movement times, higher activity frequencies, and shorter static durations than CR-Katnal2, with GF-Katnal2 + FMT2 showing the most robust recovery (Fig. 1G–N). In the light–dark test, CR-AB, GF-AB+FMT1, and GF-Katnal2 + FMT2 responded more sensitively to stimulation, whereas CR-Katnal2 showed minimal activity. GF-Katnal2 + FMT2 activity approached CR-AB levels, indicating FMT’s therapeutic effect on ASD-like zebrafish (Fig. 1O).

### Expression pattern of key genes of neuro-related pathways and SCFAs content in AB and Katnal2 zebrafish with FMT treatment

**Figure 2:**
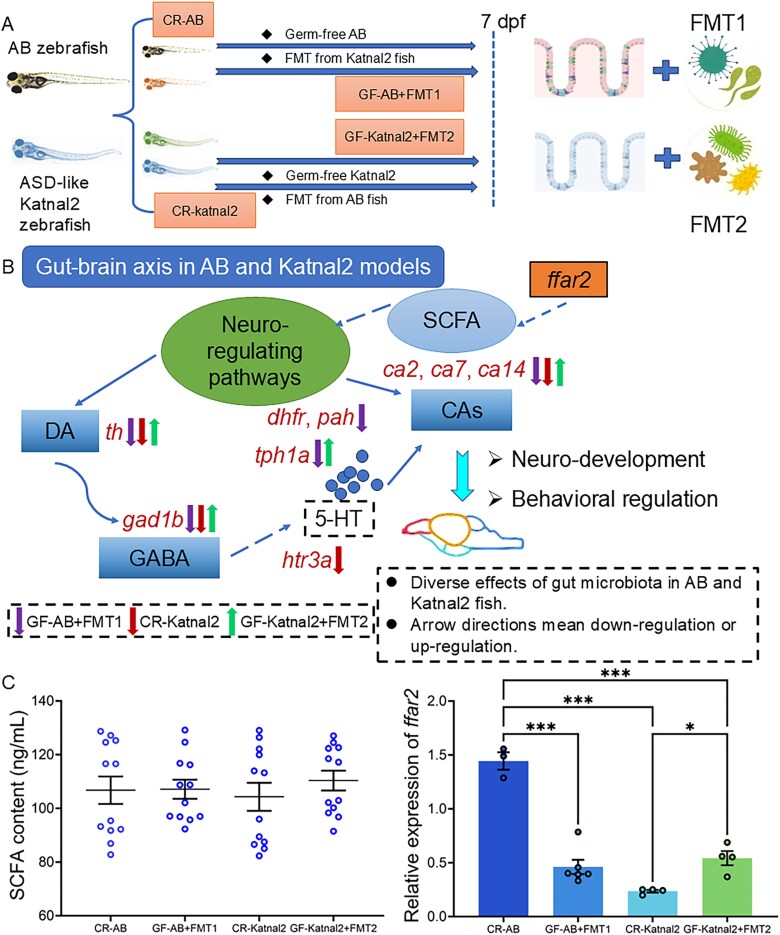
Expression pattern of key genes and SCFAs content in zebrafish after FMT between AB and Katnal2 lines. (A). Schematic scheme of the experiment. Zebrafish were generated with the GF models, and then larvae were treated with FMT between AB and Katnal2 lines. (B) Expression pattern of brain development genes: *dhfr*, *pah*; neurotransmitter genes: *tph1a*, *htr3a*, *th*; GABA-related *gad1b*; carbonic anhydrases *ca2*, *ca7*, *ca4a*, *ca14*. (C) SCFA content (ng/ mL), and *ffar2* gene expression. Each experimental group included three replicate samples, with each sample containing 30 zebrafish. Differences were considered statistically significant at ^*^P < .05, ^**^P < .01, and ^***^P < .01 when comparing the groups CR-AB, GF-AB+FMT1, CR-Katnal2, and GF-Katnal2 + FMT2.

To directly evaluate whether the microbiota regulation could improve the ASD-like zebrafish development to normal, the GF AB and Katnal2 larvae were subjected to FMT between the two lines (Fig. 2A). Gene expression analyses revealed significant alterations in pathways related to brain development and neurotransmission (5-HT, DA, GABA) (Fig. 2B and Fig. S1). Among developmental genes, *spra* was unchanged in GF-AB+FMT1, but *dhfr* was reduced (*P <* .05); GF-Katnal2 + FMT2 also showed lower *dhfr*, comparable to CR-Katnal-2 (Fig. S1A and B). *pah* was significantly lower in GF-AB+FMT1 than in both CR-AB and CR-Katnal2. For serotonergic markers, *tph1a* was downregulated (*P <* .01), and *htr3a* upregulated (*P <* .05) in GF-AB+FMT1 *versus* CR-AB, while GF-Katnal-2 + FMT2 showed no change (Fig. S1C–E). Dopaminergic *th* was suppressed in GF-AB+FMT1 and CR-Katnal2 compared with CR-AB (*P <* .05), but significantly upregulated in GF-Katnal2 + FMT2 *versus* CR-Katnal2 (*P <* .01) (Fig. S1F). For GABA-related genes, *gad1b* was reduced in GF-AB+FMT1 (*P <* .05), with no difference in GF-Katnal2 + FMT2 *versus* CR-Katnal2 (Fig. S1G). Carbonic anhydrases showed group-specific regulation: GF-AB+FMT1 decreased *ca4a* and *ca14* (*P <* .05), while GF-Katnal2 + FMT2 elevated the expression of *ca2*, *ca7*, and *ca14* (*P <* .05), with no change in *ca4a* (Fig. S1H–K). These findings indicated the distinct regulatory effects on neuro-related key genes of FMT from AB and ASD-like zebrafish donors, in which the neuro-related signaling pathways were inhibited, but the normal microbiota rescued Katnal2 fish (Fig. 2B).

Total content of SCFAs were quantified across experimental groups (Fig. 2C). No statistically significant differences were observed among the CR-AB (106.76 ng/mL), GF-AB+FMT1 (107.11 ng/mL), CR-Katnal2 (104.29 ng/mL), and GF-Katnal2 + FMT2 (110.34 ng/mL) groups, although a increasing trend was noted in the GF-Katnal2 + FMT2 group compared to CR-Katnal2. However, to indirectly assess SCFA levels, the *ffar2* gene expression was analyzed. Compared with the CR-AB group, the GF-AB+FMT1, CR-Katnal-2, and GF-Katnal2 + FMT2 groups exhibited marked downregulation of *ffar2* (Fig. 2C). GF-Katnal2 + FMT2 larvae showed significantly higher *ffar2* expression than CR-Katnal2 zebrafish, indicating potential regulatory effects of transplantation with AB zebrafish fecal solution.

### Characteristics and infection of *A. muciniphila* on AB and Katnal2 zebrafish

**Figure 3:**
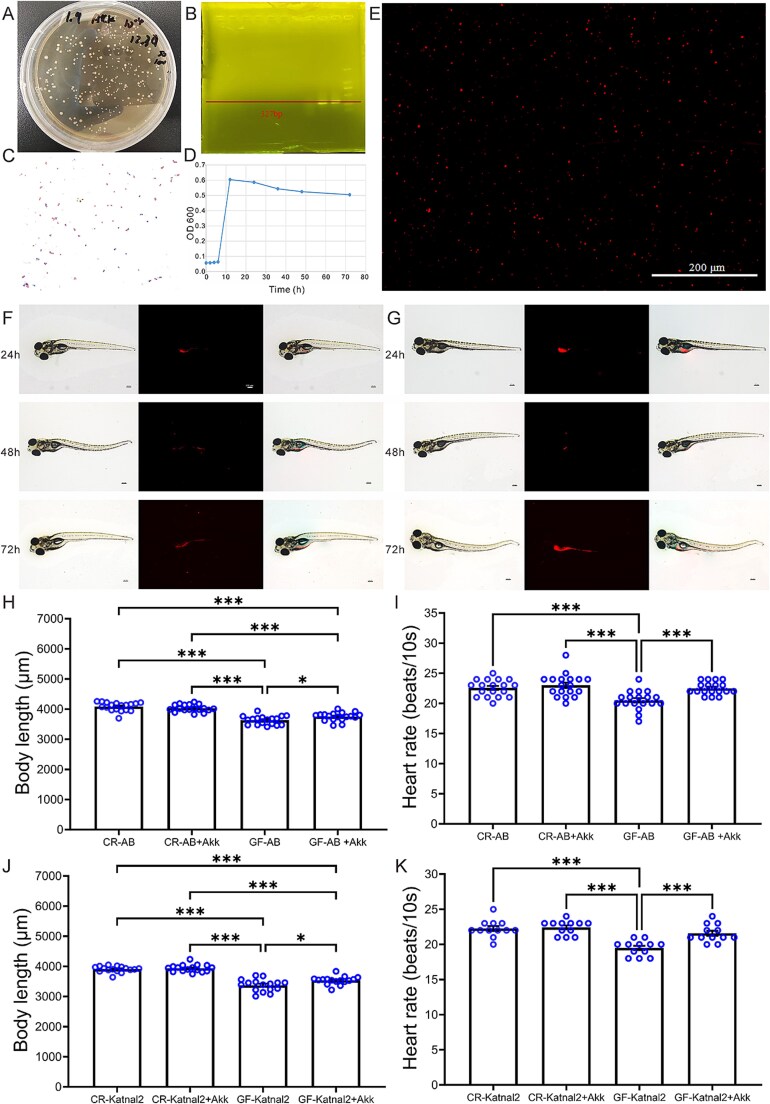
Culture, identification, colonization, and effects of *A. muciniphila* on AB and Katnal2 zebrafish development. (A) Colony morphology on mucin agar. (B) PCR amplification. (C) Gram staining. (D) Growth curve. (E) Fluorescence staining *in vitro*. Colonization in AB (F) and Katnal2 (G) intestines. Images were acquired every 24 h from 5 to 7 dpf. (H-I, J-K) Body length and heart rate of AB and Katnal2 fish. ^*^P < .05, ^**^P < .01, ^***^P < .001 indicated the significance between treat groups and the corresponding control groups.

Under anoxic conditions at 37°C, *A. muciniphila* formed milky-white colonies on mucin-supplemented BHI agar within 24 h (Fig. 3A). DNA extracted from the strain produced a distinct 327 bp band on agarose gel electrophoresis (Fig. 3B), and Gram staining confirmed its oval, Gram-negative morphology (Fig. 3C). Growth monitoring by absorbance revealed entry into the exponential phase at ~10 h (Fig. 3D). CM-DiI staining produced granular red fluorescence (Fig. 3E), enabling in vivo tracking. In zebrafish, intestinal fluorescence increased at 24 h, declined by 48 h, and persisted through 72 h, with signals progressing from the gastrointestinal tract to the intestines and cloaca, confirming colonization and persistence of the probiotic (Fig. 3F and G).

### Developmental indices of AB and Katnal2 zebrafish after *a. muciniphila* intervention

Developmental indices were assessed in CR-AB, CR-AB+Akk, GF-AB, and GF-AB+Akk zebrafish to evaluate the effects of *A. muciniphila* intervention (Fig. 3). Hatching rates at 48 hpf and body weight did not differ significantly among groups, indicating that sterilization and *A. muciniphila* exerted minimal impact on these two parameters (Fig. S2A and B). Body length was significantly shorter in GF-AB (3628.8 μm) and GF-AB+Akk (3749.2 μm) than in CR-AB (4082.33 μm, *P <* .001), although GF-AB+Akk fish were longer than GF-AB (*P <* .05), indicating partial developmental recovery (Fig. 3H). Heart rate decreased in GF-AB (20.5 beats/10 s) compared with CR-AB (22.6 beats/10 s, *P <* .001), but was restored in GF-AB+Akk (22.5 beats/10 s), which was significantly higher than GF-AB (*P <* .001) and comparable to CR-AB+Akk (23.0 beats/10 s) (Fig. 3I). While, developmental indices of CR-Katnal2, CR-Katnal2 + Akk, GF-Katnal2, and GF-Katnal2 + Akk zebrafish showed the *A. muciniphila* effects on ASD-like animal models (Fig. 3). Hatching rates at 48 hpf and body weights were not differences among groups, indicating no influence of sterilization or *A. muciniphila* treatment on these two basic aspects (Fig. S2C and D). In contrast, body length was significantly reduced in GF-Katnal2 (3370.4 μm) and GF-Katnal2 + Akk (3533.6 μm) compared with CR-Katnal2 (3906.8 μm, *P <* .001). However, *A. muciniphila* supplementation partially rescued the growth of GF-Katnal2 + Akk fish, which appeared significantly longer than GF-Katnal-2 (*P <* .01) (Fig. 3J). GF Katnal2 mutants exhibited a significantly reduced heart rate (19.5 beats/10 s) compared to their CR counterparts (22.3 beats/10 s; *P <* .001). This deficit was significantly ameliorated by GF-Katnal2 + Akk, which restored the heart rate to 21.6 beats/10 s, a value comparable to the control baseline (Fig. 3K).

### Behavioral changes of AB and Katnal2 zebrafish after *A. muciniphila* treatment

**Figure 4:**
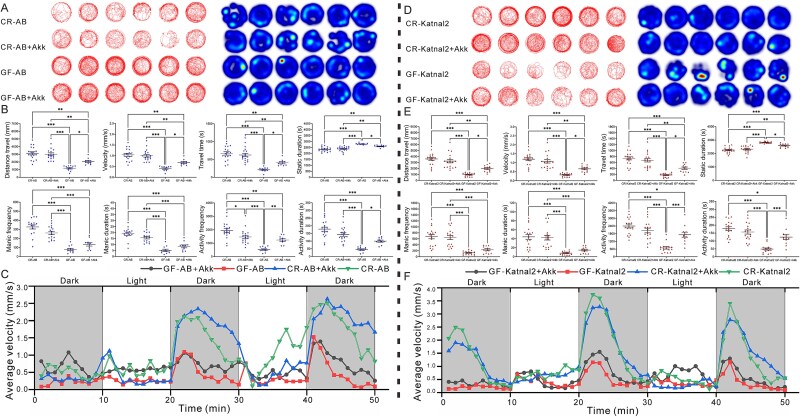
Behavioral analysis of CR and GF AB and Katnal2 zebrafish after *A. muciniphila* treatment. AB zebrafish groups in order of CR-AB, CR-AB+Akk, GF-AB, GF-AB+Akk: (A) trajectory plots and heat map. (B) Traveled distance, velocity, travel time, static duration, manic frequency, manic duration, activity frequency, activity duration. (C) Average velocity in light–dark tests. Katnal2 zebrafish groups in order of CR-Katnal2, CR-Katnal2 + Akk, GF-Katnal2, GF-Katnal2 + Akk: (D) trajectory diagram and heat map. (E) Traveled distance, velocity, travel time, cumulative duration of static, manic frequency, cumulative duration of manic, activity frequency, cumulative duration of activity. (F) Average velocity (mm/s) in the light–dark stimulation test. Data are presented from 18 zebrafish per group, with statistical significance denoted as ^*^P < .05, ^**^P < .01, and ^***^P < .001 for comparisons between groups.

Behavioral performance of AB zebrafish and *A. muciniphila*-treated larvae was assessed at 7 dpf (Fig. 4). CR-AB+Akk fish displayed stable edge-oriented trajectories similar to CR-AB, whereas GF-AB and GF-AB+Akk exhibited weaker, disordered movements with reduced activity and prolonged dwell times; however, GF-AB+Akk showed greater mobility, higher activity intensity, and shorter static periods than GF-AB (Fig. 4A). Traveled distance was unchanged in CR-AB+Akk *versus* CR-AB, but significantly reduced in GF-AB and GF-AB+Akk, with GF-AB+Akk covering more distance than GF-AB (Fig. 4B). Velocity and travel time followed similar patterns: both were reduced in GF-AB and partially restored in GF-AB+Akk, while CR-AB+Akk remained comparable to CR-AB (Fig. 4B). Static duration increased in GF groups, with GF-AB+Akk trending lower than GF-AB. Hyperactivity measures were also impaired: manic frequency and duration were the lowest in GF-AB, but both were significantly improved in GF-AB+Akk (Fig. 4B). Likewise, activity frequency and duration were strongly reduced in GF-AB but significantly higher in GF-AB+Akk, indicating the positive effects of probiotic on fish with partial behavioral rescue (Fig. 4B). In light–dark stimulation experiments, all groups increased velocity due to light off and decreased under light on; CR-AB+Akk showed the highest light-induced activity, followed by CR-AB, GF-AB+Akk, and GF-AB (Fig. 4C).

In the behavioral analyses of *A. muciniphila*-treated Katnal2 larvae (Fig. 4D), CR-Katnal2 + Akk fish exhibited gentle changes in movement trajectories compared to CR- Katnal2, whereas GF-Katnal2 and GF-Katnal2 + Akk displayed reduced and scattered movement patterns characterized by chaotic trajectories, decreased activity levels, and prolonged stays at specific locations. GF- Katnal2 + Akk showed denser movement patterns, increased trajectory density, enhanced activity levels, and shorter static than GF-Katnal2 (Fig. 4D). Traveled distance and velocity in CR-Katnal2 + Akk were alike to CR-Katnal2 fish, but significantly reduced in GF-Katnal2 and GF-Katnal2 + Akk, with Akk treatment partial recovery (Fig. 4E). Similarly, the moved time was less in GF-AB and GF-Katnal2 + Akk than CR-Katnal2, though Akk supplement exceeded GF-Katnal2 (Fig. 4E). Static duration increased in both GF groups, that of supplemented with Akk bacteria group was significant shorter static duration than other groups (Fig. 4E). Hyperactivity measures were impaired in GF-Katnal2, but GF-Katnal2 + Akk showed significantly higher manic frequency and activity frequency/duration than GF-Katnal2, indicating the absence of microbiota adverse effects and probiotic-mediated behavioral rescue (Fig. 4E). In light–dark testing, all groups increased velocity under dark and decreased with light on; CR-Katnal2 + Akk showed the strongest response to light–dark stimulation, followed by CR-Katnal2, GF-Katnal2 + Akk, and GF-Katnal2 (Fig. 4F).

### Changes of microbiota of AB and Katnal2 zebrafish before and after *A. muciniphila* treatment

**Figure 5:**
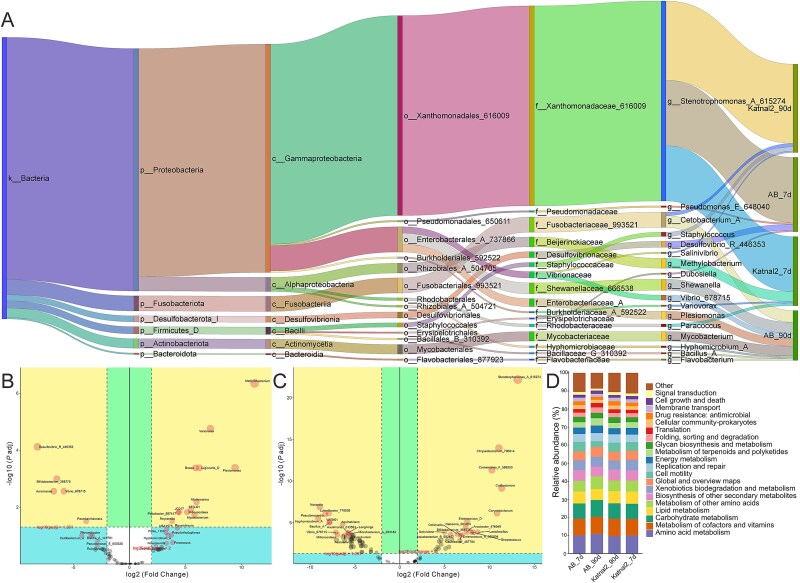
Comparison of microbiota composition and predicted functions in AB and Katnal2 zebrafish larvae and adults. (A) Basic dynamic Sankey of species abundance between AB and Katnal2 at different life stages. (B, C) DESeq2 volcano of CR-AB vs. CR-Katnal2 at 7d and 90d stages. (D) Predicted KEGG functions of microbiota in four groups based on sequence percentages (%).

To discover the differences between ASD-like animal models and controls, composition of gut microbiota in AB and Katnal2 zebrafish larvae (7d) and adult (90d) was profiled by 16S rRNA genes (V3–V4) sequencing (Fig. 5). The α-diversity analysis (Shannon, Simpson, observed ASVs) revealed significantly greater diversity and abundance in adult AB zebrafish compared with Katnal2 (Table S1). The principal coordinate analysis (PCoA) clearly separated the two groups (Fig. S3A), and ASV analysis showed 504 features in AB and 209 in Katnal2, with 46 shared (Fig. S3B). At the phylum level, Proteobacteria dominated Katnal2 (87.67%) but were less abundant in AB (39.57%), whereas *Firmicutes*, *Actinobacteria*, *Fusobacteriota*, and *Bacteroidota* were enriched in AB (Fig. 5A and Fig. S3C). At the genus level, *Stenotrophomonas* was strikingly higher in Katnal2 (84.23% vs. 0.09% in AB), while *Cetobacterium*, *Shewanella*, *Mycobacterium*, *Plesiomonas*, and *Vibrio* were all more abundant in AB (Fig. 5A and Fig. S3D). LEfSe analysis (LDA > 2, *P <* .05) confirmed distinct microbial signatures: AB zebrafish were enriched in *Mycobacterium*, *Shewanella*, *Vibrio*, and *Enterobacterales*, whereas Katnal2 were dominated by *Stenotrophomonas*, *Chryseobacterium*, and *Comamonas* genera (Fig. 5B and Fig. S3E and F). These findings highlighted profound microbiota differences between AB and Katnal2 zebrafish, suggesting potential microbial contributions to ASD-related phenotypes.

At 7 dpf, α-diversity indices showed minimal differences across groups (Table S1), and PCoA revealed no clear separation between AB and Katnal2 larvae (Fig. S4A). Venn analysis identified 843 features in AB and 246 in Katnal2, with 97 shared (Fig. S4B). Taxonomic profiling of the top 20 bacterial taxa showed Proteobacteria as the dominant phylum in both groups (88.1% in AB, 95.4% in Katnal2), with Bacteroidota and Actinobacteria relatively enriched in Katnal2, while Firmicutes and Desulfobacterota were more abundant in AB (Fig. 5A and Fig. S4C). At the genus level, *Stenotrophomonas*, *Methylobacterium*, *Desulfovibrio*, *Paracoccus*, and *Pseudomonas* accounted for >70% of the microbiota in both groups (Fig. 5A and Fig. S4D). AB larvae were enriched in *Stenotrophomonas* (69.5%), *Desulfovibrio* (6.4%), and *Pseudomonas* (1.6%), whereas Katnal2 larvae showed higher levels of *Methylobacterium* (10.4%) and *Paracoccus* (2.8%). LEfSe analysis further highlighted *Bosea* and *Variovorax* as discriminant taxa in Katnal2 larvae (Fig. 5C and Fig. S4E and F).

DESeq2 analysis revealed 12 significantly upregulated taxa (e.g. *Methylobacterium*) and 5 downregulated taxa (e.g. *Bifidobacterium*) in the larval CR-AB *vs*. CR-Katnal2 comparison. *Variovorax* and *Desulfovibrio* exhibited the most pronounced changes in abundance (Fig. 5B). The significantly upregulated taxa (e.g. *Stenotrophomonas*; *Chryseobacterium*) and downregulated taxa (e.g. *Nocardia*; *Luteolibacter*) were discovered in the adult CR-AB vs. CR-Katnal2 groups (Fig. 5C). Additionally, KEGG functions of microbiota were predicted, and distinct functional shifts among treatment groups were revealed. For example, Amino acid and Carbohydrate metabolism were elevated in AB from 7d to 90d but not in the Katnal2 period. Replication and repair, and Translation showed increased trends in both fish lines, while Drug resistance: antimicrobial was opposite in two lines, suggesting altered core metabolic pathways (Fig. 5D).

### Reshaped gut microbial composition in AB and Katnal2 zebrafish by *A. muciniphila* treatment

**Figure 6:**
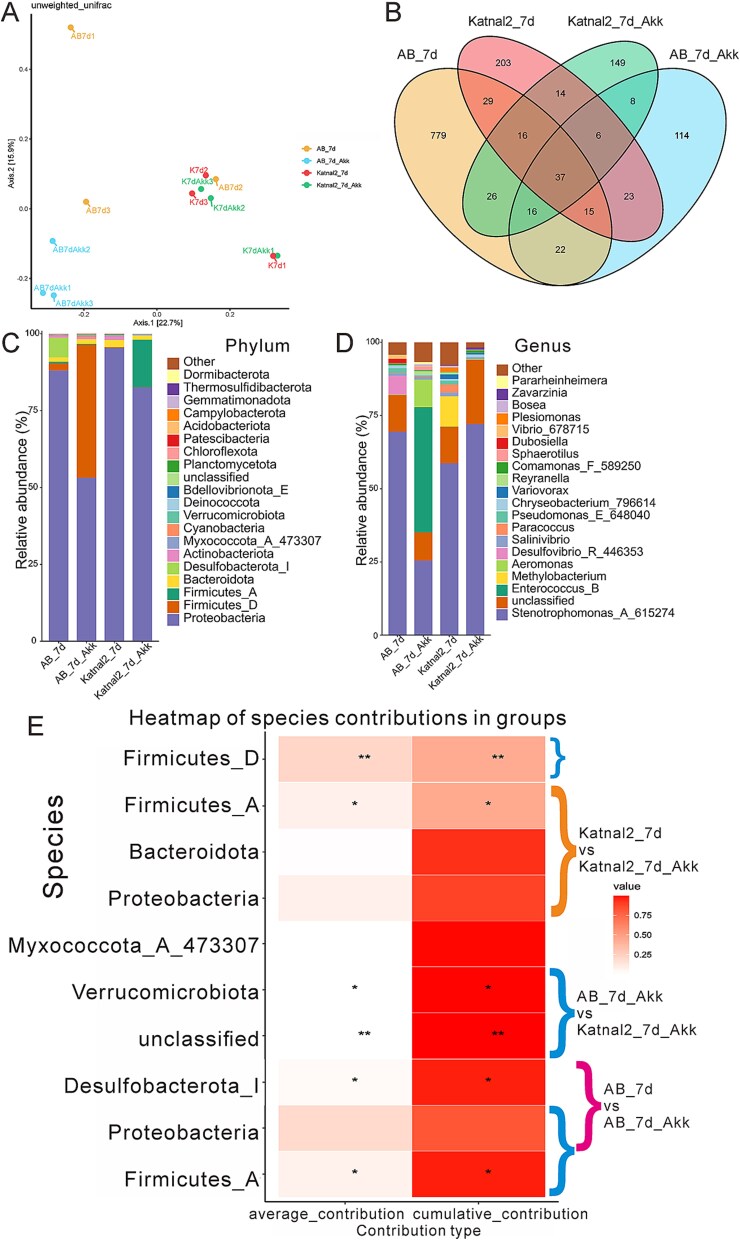
Microbiota composition in larval AB and Katnal2 zebrafish after *A. muciniphila* treatment. (A) PCoA. (B) Venn diagram. (C, D) Phylum- and genus-level composition (n = 3). (E) Heat map of species with the greatest contributions to explore community structure differences by Simper analysis in CR-AB vs. CR-AB+Akk, CR-Katnal2 vs. CR-Katnal2 + Akk, and CR-AB+Akk vs. CR-Katnal2 + Akk, which were indicated by red, yellow, and blue color brackets.

16S rRNA genes sequencing revealed that *A. muciniphila* colonization did not alter overall α-diversity or PCoA clustering (Table S1, Fig. 6A) but shifted microbial composition. In AB fish, Venn analysis showed 850 vs. 151 features in CR-AB and CR-AB+Akk, with 90 shared (Fig. 6B). Among which, Firmicutes phylum, *Enterococcus,* and *Aeromonas* genera were enriched, while Proteobacteria phylum, *Stenotrophomonas*, and *Desulfovibrio* declined after *A. muciniphila* intervention (Fig. 6C and D). In Katnal2 larvae, 16S rRNA genes analysis detected 270 and 199 microbial features in the CR-Katnal2 and CR-Katnal2 + Akk groups, respectively, with 73 features shared between them (Fig. 6B). Probiotic treatment shifted the microbial composition, increasing the relative abundance of Firmicutes while decreasing that of Proteobacteria (Fig. 6C). At the genus level, this was characterized by an increase in Stenotrophomonas and decreases in *Methylobacterium* and *Paracoccus* (Fig. 6D). Further, Simper analysis proved the species that most contributed to the community structures differences in CR-AB vs. CR-AB+Akk, CR-Katnal2 vs. CR-Katnal2 + Akk, and CR-AB+Akk vs. CR-Katnal2 + Akk sets, which highlighted the dynamic alteration and roles of *Desulfobacterota*, *Firmicutes_A*, *Firmicutes_D*, and *Verrucomicrobiota* after probiotic treatment (Fig. 6E). Developmental comparisons further showed distinct stage-specific shifts in AB and Katnal2 zebrafish, with larvae dominated by Proteobacteria and taxa, such as *Stenotrophomonas*, *Reyranella*, and *Pseudomonadaceae*, while adults were enriched in *Firmicutes*, *Fusobacteriota*, and genera including *Cetobacterium*, *Shewanella*, *Mycobacterium*, *Bifidobacterium*, and *Enterococcus* (Fig. S5 and Fig. S6). Together, these results demonstrated that while diversity remained stable, *A. muciniphila* selectively reshaped microbial composition in a lineage- and stage-dependent manner.

### Effects of *A. muciniphila* intervention on the expression of neuro-regulation genes in AB zebrafish

**Figure 7:**
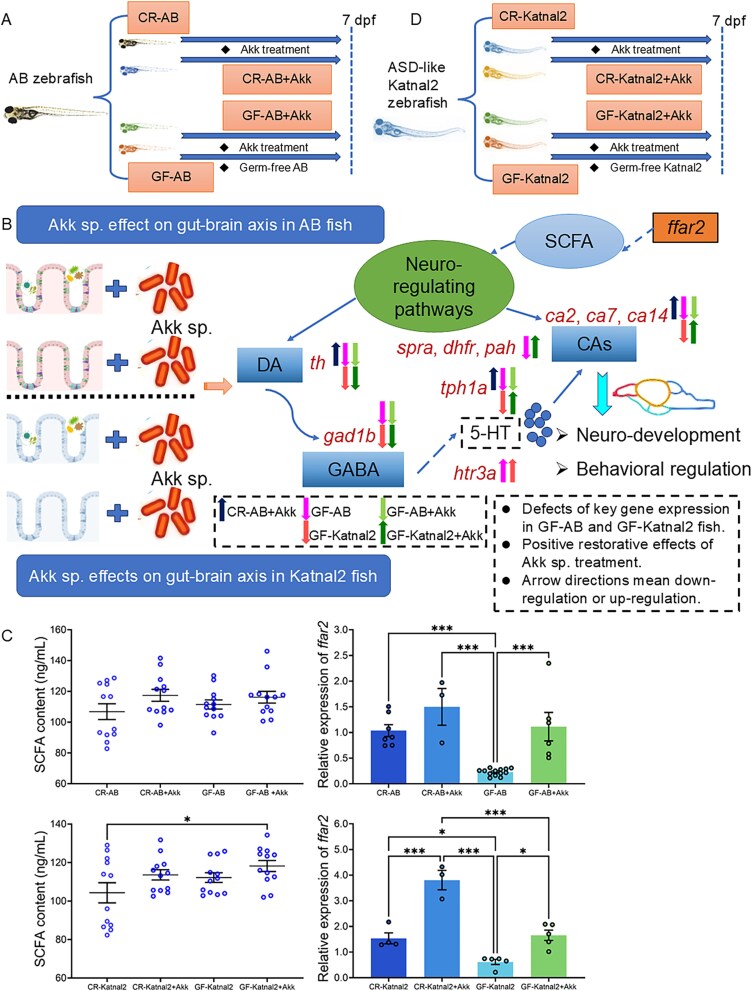
Expression pattern of key genes and SCFAs content in AB and Katnal2 zebrafish larvae after *A. muciniphila* treatment. (A, D). Schematic scheme of the experiment. Zebrafish were generated with the GF models, and then larvae were treated with probiotics under both CR and GF conditions. (B) Expression pattern of brain development genes in AB and Katnal2 fish groups: *dhfr*, *pah*; neurotransmitter genes: *tph1a*, *htr3a*, *th*; GABA-related *gad1b*; carbonic anhydrases *ca2*, *ca7*, *ca4a*, *ca14*. (C) SCFAs content (ng/mL), and *ffar2* gene expression in AB and Katnal2 fish groups. Data are based on triplicate samples per group, with each sample comprising 30 fish. Statistical significance (denoted as ^*^P < .05, ^**^P < .01, ^***^P < .001) applies to separate comparisons between the indicated groups: within the AB lineage (CR-AB, CR-AB+Akk, GF-AB, GF-AB+Akk) or within the Katnal2 lineage (CR-Katnal2, CR-Katnal2 + Akk, GF-Katnal2, GF-Katnal2 + Akk).

Treatment with *A. muciniphila* enhanced activity in GF-AB larvae, suggesting modulation of brain development *via* the microbiota-gut-brain axis and neurotransmitter involving serotonin (5-HT), dopamine (DA), and gamma-aminobutyric acid (GABA) (Fig. 7). Expression of brain-development genes *pah* and *dhfr* was altered, compared with CR-AB, the CR-AB+Akk group showed no changes in *spra*, *pah*, or *dhfr*, whereas both GF-AB and GF-AB+Akk exhibited significantly reduced *pah* expression (*P <* .01, .001) (Fig. S7A–C), indicating limited effects of *A. muciniphila* on AB brain development. For the 5-HT system, *tph1a* was suppressed in GF-AB (*P <* .001) but upregulated in CR-AB+Akk (*P <* .05), while *htr3a* was increased in GF-AB (*P <* .05) and downregulated in GF-AB+Akk (Fig. S7D and E). Dopaminergic *th* was elevated in CR-AB+Akk *versus* CR-AB (*P <* .05) (Fig. S7F). GABA-related *gad1b* was significantly inhibited in both GF-AB and GF-AB+Akk (*P <* .001) (Fig. S7G). Carbonic anhydrases showed divergent regulation: CR-AB+Akk upregulated *ca2*, *ca4a*, *ca7*, and *ca14* (all *P <* .05), whereas GF-AB reduced *ca2* and *ca14* (*P <* .01), and GF-AB+Akk further suppressed *ca2* (*P <* .01) and *ca14* (*P <* .001), with no significant differences between GF-AB and GF-AB+Akk (Fig. S7H–K). These results indicated that the probiotic promoted neuro-related signaling pathways in CR-AB fish, while the GF-AB fish showed significantly suppressed and gently improved by *A. muciniphila* intervention (Fig. 7B). Whole-body SCFAs content did not differ significantly among groups: CR-AB zebrafish (106.8 ng/mL), CR-AB+Akk (117.4 ng/mL), GF-AB (111.5 ng/mL), and GF-AB+Akk (116.2 ng/mL) (Fig. 7C). However, compared with the CR-AB group, the GF-AB group exhibited a significant decrease in *ffar2* gene expression, whereas the GF-AB+Akk group increased the *ffar2* expression relative to GF-AB (Fig. 7C).

### Modulated neuro-regulatory gene expression in Katnal2 zebrafish by *A. muciniphila* intervention

In terms of expression of neurodevelopment- and neurotransmitter-related genes in Katnal2 zebrafish, *A. muciniphila* supplementation did not alter *spra*, *pah* (Fig. 7D, Fig. S8), or *dhfr* expression in CR-Katnal2 + AKK fish. However, GF-Katnal2 + Akk fish elevated *spra* expression (*P <* .01) and, relative to GF-Katnal2, higher *spra* (*P <* 0.001) and *dhfr* (*P <* .05) levels (Fig. S8A–C). The GF-Katnal2 group showed a lower level of *tph1a* (*P <* .01) compared with CR-Katnal2, whereas *A. muciniphila* intervention restored its expression (*P <* .001) (Fig. S8D). In contrast, *htr3a* was upregulated in GF-Katnal2 (*P <* .001) but suppressed by *A. muciniphila* (*P <* .01) (Fig. S8E). Expression of *th* was inhibited in both GF-Katnal2 (*P <* .01) and GF-Katnal2 + Akk (*P <* .05) than CR-Katnal2 (Fig. S8F). No changes were observed in *gad1b* (Fig. S7G). *A. muciniphila* supplementation increased carbonic anhydrase expression, including *ca2* (*P <* .01), *ca4a* (*P <* .05), *ca7* (*P <* .001), and ca14 (*P <* .05), in GF-Katnal2 fish (Fig. S8H–K), suggesting enhanced GABAergic neuronal activity and potential regulatory effects of bacteria on ASD-like zebrafish. These findings proved that the probiotic displayed no different changes on the neuro-related signaling pathways in CR-Katnal2 fish, but the GF-Katnal2 fish showed significant inhibition and rescued by *A. muciniphila* intervention (Fig. 7B). Furtherly, SCFA content in Katnal2 zebrafish was 104.2 ng/ml, 113.6 ng/ml, 112.2 ng/ml, and 118.2 ng/ml across the four groups, with the GF-Katnal2 + Akk group significantly higher than the CR-Katnal2 fish (Fig. 7C). Expression of the gene *ffar2* was used as an indirect readout of SCFA levels: compared with CR-Katnal2, the CR-Katnal2 + Akk group showed a significant increase (Fig. 7C). The GF-Katnal2 group significantly reduced *ffar2* expression, whereas GF-Katnal2 + Akk zebrafish exhibited restored *ffar2* expression to near-normal CR-Katnal2 levels following *A. muciniphila* intervention, indicating a partial restorative effect of the probiotic.

### Correlation analysis between gut microbiota and key genes, developmental and behavioral indexes of diverse zebrafish models

**Figure 8:**
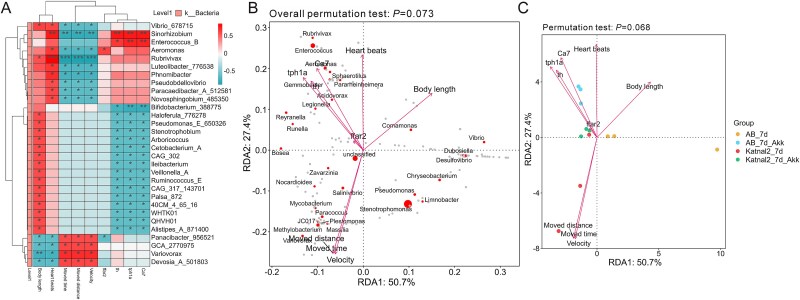
Correlation analysis of gut microbiota with key genes, developmental indices, and behavioral abilities in zebrafish after probiotic treatment. (A) Spearman correlation analysis of traits between gut microbiota and key genes, developmental indices, and behavioral abilities. (B) Redundancy analysis (RDA) by permutation test of environmental factors including heartbeats, body length, moved distance, moved time, velocity, and *th*, *tph1a, ca7, ffar2* expression. (C). Relationship between microbial features and environmental factors in groups of CR-AB, CR-AB+Akk, CR-Katnal2, and CR-Katnal2 + Akk. ^*^P < .05, ^**^P < .01, ^***^P < .001.

Spearman analysis revealed a significant correlation between gut microbiota and host physiological and behavioral traits. *Vibrio*, *Sinorhizobium*, *Enterococcus*, and *Aeromonas* showed strong positive correlations with DEGs and heartbeats. In contrast, *Devoisia* and *Variovorax* exhibited negative correlations with developmental indexes but positive correlations with behavioral ability. Clustering analysis grouped microbiota related to gene expression, movement, and development, suggesting their potential roles in promoting host vitality (Fig. 8A). Redundancy analysis (RDA) by permutation test (*P* = .073, RDA1 = 50.7%, RDA2 = 27.4%) of key factors revealed that *Aeromonas*, *Enterococcus*, and *Gemmobacter* were positively correlated with heart beats and *th*, *tph1a, ca7, ffar2* expression, while *Vibrio* and *Desulfovibrio* were associated with body length, *Mycobacterium*, and *Plesiomonas* were closely related to moved distance, moved time, velocity (Fig. 8B). Relationship between microbial features and traits with permutation test indicated marginal significances (*P =* .068) in groups of CR-AB, CR-AB+Akk, CR-Katnal2, CR-Katnal2 + Akk. Samples within the distinct CR-AB clusters showed a positive correlation with heart rate, whereas those in the CR-Katnal2 clusters were associated with velocity. Key features (e.g. *ffar2*, *th*, *tph1a*, *ca7*) exhibited sample-specific distributions in CR-AB+Akk and CR-Katnal2 + Akk groups, demonstrating positive regulation of *A. muciniphila* on host microbial remolding and the subsequent physiological functions (Fig. 8C).

## Discussion

ASD is a complex neurodevelopmental condition whose etiology and pathophysiological mechanisms are not fully understood. Evidence points to diverse contributing factors, including immune dysregulation, oxidative stress, gut microbiome alterations, and hormonal imbalances [40, 41]. Accumulating evidence implicates the gut-brain axis, particularly the gut microbiota, as a critical modulator of ASD risk and symptom severity [42]. In this study, we employed Katnal2 mutant zebrafish, a model of ASD-like phenotypes, to investigate the interplay between host genetics, microbial communities, and neurodevelopmental outcomes. GF zebrafish were used to investigate the role of gut microbiota in neurodevelopmental disorders. GF fish exhibited impaired development and behavioral deficits compared to CR counterparts. Our findings provide compelling evidence that gut microbiota composition and specific bacterial taxa profoundly influence growth, behavior, and neuro-regulatory pathways, as demonstrated in GF models and through FMT between AB and Katnal2 lines.

Katnal2 mutants exhibited marked developmental delays, including reduced hatching rates, body length, body weight, and heart rate, consistent with impaired growth trajectories [13]. FMT experiments revealed a bidirectional influence of microbial communities on host phenotypes [43]. In this study, transplantation of microbiota from Katnal2 donors into GF-AB recipients induced growth retardation and behavioral abnormalities, whereas microbiota from AB donors transplanted into GF-Katnal2 recipients ameliorated these deficits. These GF fish, with their sterile microbial background, serve as reliable models for investigating the specific roles of gut microbiota through controlled FMT and bacterial colonization studies. While FMT from conventionally reared donors positively affected GF-Katnal2 larvae, future studies could consider additional control groups, such as GF fish without FMT, or employ autologous FMT designs [44]. Previously, dysbiosis and altered microbial metabolites were frequently reported in children with autism [45], and FMT improved gastrointestinal and neuropsychiatric symptoms [24]. Consistent with these reports, our findings in Katnal2 mutants underscore a causal role for gut microbiota in modulating ASD-like phenotypes.

Behavioral analyses further supported this conclusion. Microbiota from Katnal2 donors reduced locomotor activity, increased static time, and impaired responses to light–dark stimulation in AB recipients. Conversely, microbiota from AB donors enhanced locomotor activity and reduced behavioral abnormalities in Katnal2 recipients. These results suggest that microbial communities influence not only somatic development but also neurobehavioral outcomes directly. However, zebrafish larvae may not fully replicate the behavioral complexity of human ASD, particularly deficits in social communication. Future studies employing chronic treatments and behavioral evaluations in adult zebrafish are warranted.

Further, in the central nervous system, key pathways of GABA, DA, and 5-HT potentially regulate stress responses, anxiety, and aggressive behaviors by neurochemical and metabolic patterns [46]. At the molecular level, we observed pronounced alterations in genes associated with neurotransmitter systems. Katnal2-derived microbiota suppressed the brain development-related genes *pah*, *dhfr*, and *htr3a* (serotonin receptor), but significantly elevated *tph1a* expression. In the GF-AB+FMT1 group, both *th* (DA pathway, dopamine synthesis) and *gad1b* (GABA synthesis) were obviously decreased. Conversely, GF-Katnal2 + FMT2 with transplantation from AB zebrafish increased *th*, *gad1b*, and carbonatease (*ca2*, *ca4a*, *ca7*, *ca14*) levels. These findings reveal the altered serotonin and dopamine signaling in ASD-like zebrafish, especially the microbiota-loss models (GF), highlighting the capacity of gut microbiota to regulate central neurotransmission. Similarly, the role of the microbiota–gut–brain axis in pediatric populations is underscored by a multi-omics study in children and adolescents with major depressive disorder (MDD). Transplantation of fecal microbiota from these patients into adolescent rats induced depressive-like behaviors, highlighting how gut microbiota alterations can inform therapeutic strategies for pediatric depression [47]. The observed modulation of carbonic anhydrase genes suggests additional effects on pH regulation and neuronal excitability [48], thereby further linking microbial composition to neurophysiological function. Gut bacteria alter the synthesis and degradation of neurotransmitters by the production of specific metabolic compounds, such as bile acids, short-chain fatty acids (SCFAs), GABA, DA, and 5-HT [49]. SCFAs, produced by gut bacteria, bind to free fatty acid receptors (FFARs) on intestinal epithelial cells (IECs). They can then interact with enteric neurons or enter the circulatory system, and are widely recognized as key microbial metabolites that influence brain function [23]. This study found no significant changes in SCFA levels following FMT; instead, we observed differential expression of the SCFA receptor *ffar2*, suggesting that microbial modulation of host signaling pathways, rather than absolute metabolite levels, may be critical in shaping neurodevelopmental outcomes.

The microbiota of AB zebrafish were enriched in the phylum Firmicutes and the genus *Pseudomonas*, whereas the microbiota of Katnal2 mutants were dominated by Proteobacteria and the genera *Acetobacter* and *Bacillus*. Similar microbial shifts have been reported in the feces of autistic children, including a significantly decreased Bacteroidetes/Firmicutes ratio and lower abundances of *Fusobacteria* and *Verrucomicrobia* compared to neurotypical controls [50]. These compositional differences may underlie the divergent physiological effects exerted by the microbiota on the host [51]. Similar compositional changes in the gut microbiota were observed between AB and Katnal2 zebrafish, highlighting the importance of dynamic microbiome evaluation in animal models of disease. The altered taxa (e.g. *Methylobacterium*) in Katnal2 larvae indicated the action as potential biomarkers for disease early warning. After probiotic intervention, the dynamic alteration of *Desulfobacterota*, *Firmicutes*, and *Verrucomicrobiota* appeared in AB and Katnal2 zebrafish comparative groups. Thus, these findings indicate that gut microbiota alterations and their functional consequences in ASD models, and the subsequent regulation by *A. muciniphila*, may improve autism-related symptoms and gene expression *via* microbial restructuring.

Another novelty of this study was the demonstration that supplementation with *A. muciniphila*, a next-generation probiotic, ameliorated ASD-like symptoms in the GF AB and Katnal2 zebrafish. *A. muciniphila* colonization promoted growth, increased heart rate, and enhanced locomotor activity in zebrafish larvae, while also modulating key neuro-regulatory genes and upregulating *ffar2* expression. GF-AB and GF-Katnal2 larvae exhibited the most pronounced behavioral deficits, including reduced distance traveled, movement time, velocity, manic frequency, manic duration, active frequency, and active duration during spontaneous swimming, as well as significantly slower velocity in light–dark stimulation tests. However, *A. muciniphila* treatment ameliorated these behavioral deficits in both lines, particularly when compared to their respective GF controls. These effects were most pronounced under sterile conditions, suggesting the contribution of *A. muciniphila* in reshaping microbial communities and restoring host-microbe signaling pathways. *A. muciniphila* intervention remodeled the intestinal microbiota of both AB and Katnal2 zebrafish, reducing Proteobacteria and increasing Firmicutes taxa. Additionally, a recent pilot study illustrated the effects and potential mechanisms of probiotic intervention in ASD, showing modulation of the gut microbiome and related metabolic pathways (such as SCFA and GABA pathways) involved in the gut–brain axis [52]. Correlation analysis revealed that the major developmental indices, behavioral measures, and gene expression (*ffar2*, *th*, *ca7*) were closely related to the shifted genera (*Aeromonas*, *Enterococcus*, *Gemmobacter*, *Vibrio*, *Desulfovibrio*, *Mycobacterium*, and *Plesiomonas*) in comparison of AB and Katnal2 zebrafish. Samples from the two fish lines showed distinct, positive correlations with different key features, further suggesting that microbial composition and its neuro-modulatory impacts are driven by both genetic background and probiotic intervention. Thus, these findings elucidate key interactions within the gut microbiota and demonstrate rescue of signaling pathway gene expression in Katnal2 and GF zebrafish, highlighting the potential of targeted microbial interventions for neurodevelopmental disorders.

Taken together, our results demonstrated that gut microbiota play a causal role in shaping growth, behavior, and neuro-regulation in GF and ASD-like zebrafish models. The bidirectional effects of FMT between AB and Katnal2 zebrafish provided direct evidence that microbial communities could either exacerbate or alleviate ASD-like symptoms. The beneficial effects of *A. muciniphila* suggested that precise microbial modulation may represent a promising therapeutic avenue for ASD. Future studies should aim to identify the specific bacterial taxa and metabolites responsible for these effects, and to determine whether similar mechanisms operate in mammalian models and human patients. By integrating host genetics, microbial ecology, and neurobiology, this work advanced an in-depth understanding of the gut-brain axis in ASD and opened new avenues for microbiota-based interventions [53].

## Supplementary Material

SI_wrag074_new

## Data Availability

All raw 16S rRNA genes sequencing data supporting this study have been deposited in the NCBI Sequence Read Archive (SRA) and are available under the BioProject accession number PRJNA1433129 (https://www.ncbi.nlm.nih.gov/bioproject/PRJNA1433129).
