# Supplementary material for: Fecal microbiota transplantation and Akkermansia muciniphila restore neurodevelopment and behavior via the gut-brain axis in autism-like zebrafish
Source: ISME J. 2026 Mar 30;20(1):wrag074. doi: 10.1093/ismejo/wrag074 (PMC13137330; doi:10.1093/ismejo/wrag074)
Supplement: SI_wrag074_new [file si_wrag074_new.docx]

**Supplement Information**

**Fecal microbiota transplantation and *Akkermansia muciniphila* restore neurodevelopment and behavior *via* the gut-brain axis in autism-like zebrafish**

**Running title:** *Akkermansia Rescues ASD*

Pan-Pan Jia^1^, Yan Li^1^, Hao-Yu Yang^1^, Yuan Ding^1^, Feng-Yi Guo^1^, Ming-Fei Wu^1^, Jin-Qiu Jia^1^, De-Sheng Pei^1,2^*

^1^School of Public Health, Chongqing Medical University, Chongqing 400016, China

^2^Chongqing Miankai Biotechnology Research Institute Co., Ltd., Chongqing 400025, China

*Corresponding author. Chongqing Medical University, 1 Yixueyuan Road, Yuzhong District, Chongqing 400016, China. E-mail: deshengpei@gmail.com (D.S.P).

**Text S1.** **Generation of germ‑free zebrafish**. Germ‑free (GF) zebrafish were generated using an optimized protocol described previously (Jia et al., *JOVE*, 2024). Healthy embryos were selected, and non‑viable embryos were discarded. All subsequent steps were performed on a sterile bench. The detailed sterilization procedure is as follows:

1. **Initial preparation:** Fertilized eggs were transferred into a sterile culture dish containing AB‑GZM solution and incubated at 28 °C for 6–8 h.
2. **Primary washing and disinfection:** Embryos were washed three times with GZM (3 min per cycle), followed by a brief immersion in 0.04% PVP‑I for < 1 min.
3. **Chemical sterilization:** After another three washes with GZM, embryos were treated with 0.002% sodium hypochlorite for 15 min.
4. **Final rinsing and cultivation:** To remove residual sterilizing agents, embryos were rinsed thoroughly with GZM three additional times. Then, seven embryos were aseptically transferred into each well of a sterile six‑well plate containing 7 mL of fresh GZM.
5. **Maintenance and verification:** During the culture period, 50% of the GZM medium was replaced daily. The germ‑free status was confirmed by routine bacterial detection assays.

**Test S2. Bioinformatics analysis of microbiota sequencing in zebrafish lines.**

Bioinformatics analysis was performed following the QIIME 2 documentation (https://docs.qiime2.org/2019.1/) along with customized program scripts. Briefly, raw FASTQ files were imported into a format compatible with the QIIME 2 system. Demultiplexed sequences from each sample were quality filtered, trimmed, denoised, and merged, and chimeric sequences were identified and removed using the QIIME 2 dada2 plugin to generate a feature table of amplicon sequence variants (ASVs). Taxonomy was assigned using the QIIME 2 feature-classifier plugin, which aligned ASV sequences to a pre-trained Greengenes 13_8 99% database trimmed to the V3–V4 region defined by the 338F/806R primer pair. Contaminating mitochondrial and chloroplast sequences were filtered using the QIIME 2 feature-table plugin. Differential abundance analysis was performed using ANOVA, LEfSe, and DESeq2 to identify bacterial taxa with significant differences among samples and experimental groups. Alpha diversity indices, including observed ASVs and the Shannon diversity index, were calculated to estimate microbial diversity within individual samples. Beta diversity was assessed to investigate structural variation in microbial communities across samples and was visualized using principal coordinate analysis (PCoA). Spearman’s rank correlation was used to evaluate associations among predominant taxa and key physiological indices. In addition, potential functional profiles of microbial communities were predicted using PICRUSt based on KEGG Ortholog (KO) annotations.

**Table S1. The α diversity of intestinal microbiota in AB and Katnal2 zebrafish**

| **Group** | **Coverage** | **Shannon** | **Simpson** | **Observed ASVs** |
| --- | --- | --- | --- | --- |
| **Adult-AB** | 1.00±0.00 | 4.55±1.32* | 0.88±0.08* | 275.84±82.92* |
| **Adult-Katnal2** | 1.00±0.00 | 1.44±0.72 | 0.30±0.16 | 125.00±52.12 |
| **Larval-AB** | 1.00±0.00 | 2.31±.89 | 0.50±0.18 | 346.37±329.75 |
| **Larval-Katnal2** | 1.00±0.00 | 2.52±2.42 | 0.47±0.41 | 134.67±91.52 |
| **Larval-AB+Akk** | 1.00±0.00 | 2.51±0.28 | 0.54±0.09 | 102.67±18.15 |
| **Larval-Katnal2+Akk** | 1.00±0.00 | 1.73±1.16 | 0.43±0.30 | 112.00±79.83 |

Note: * indicates a significant difference between columns (*P*<0.05).


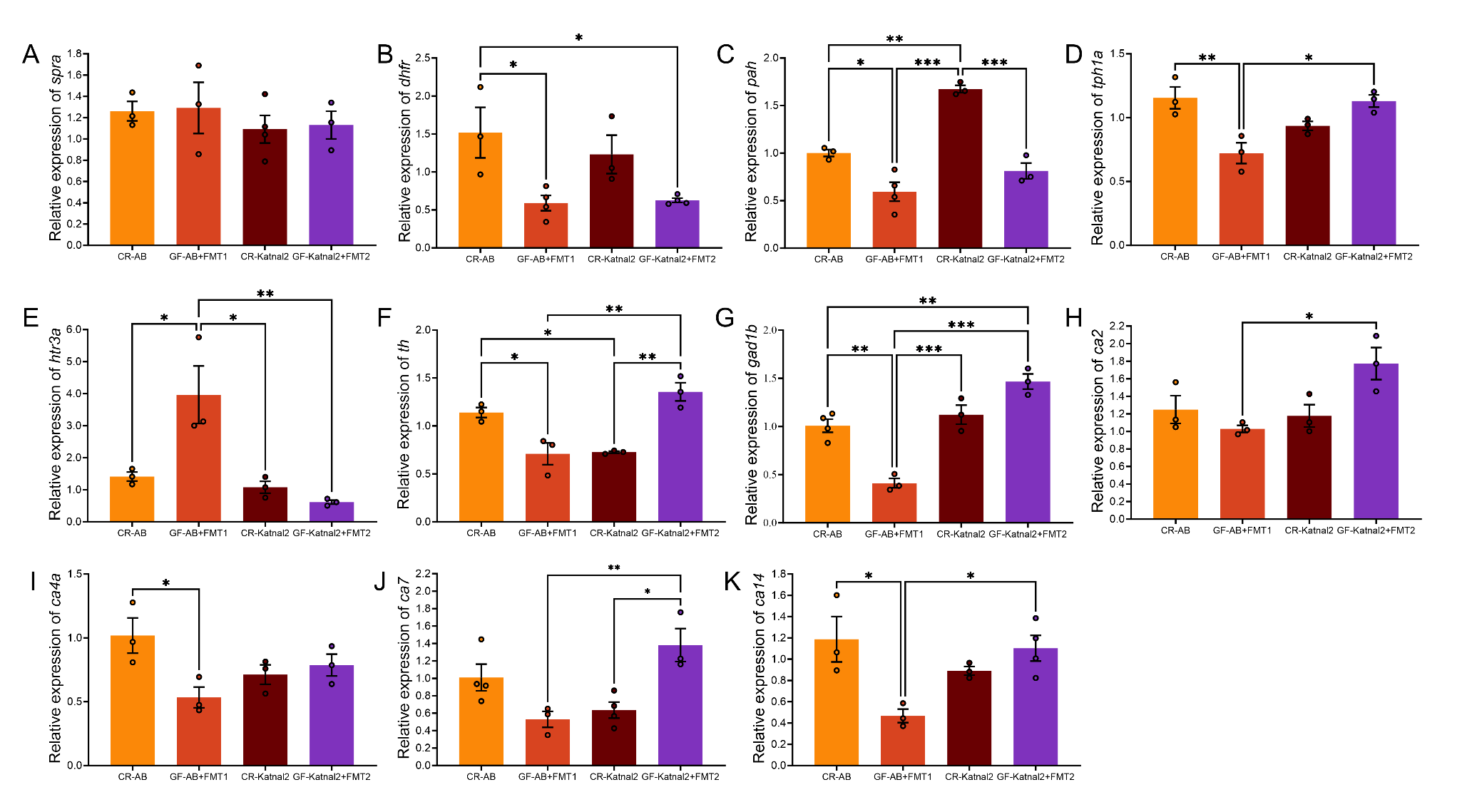


**Figure S1. Effect of FMT on the expression of key genes in AB and Katnal2 zebrafish models.** (A-C) Brain development genes: *spra*, *dhfr*, *pah*; (D-F) neurotransmitter genes: *tph1a* (D), *htr3a* (E), *th* (F); (G) GABA‑related gene gad1b; (H-K) carbonic anhydrase genes: *ca2* (H), *ca7* (I), *ca4a* (J), and *ca14* (K). Each replicate contained n = 30 zebrafish; three replicates were analyzed per group. Significance levels: **P*< 0.05, ***P*< 0.01, ****P*< 0.001 vs. CR‑AB, GF‑AB + FMT1, CR‑*Katnal2*, GF‑Katnal2 + FMT2.


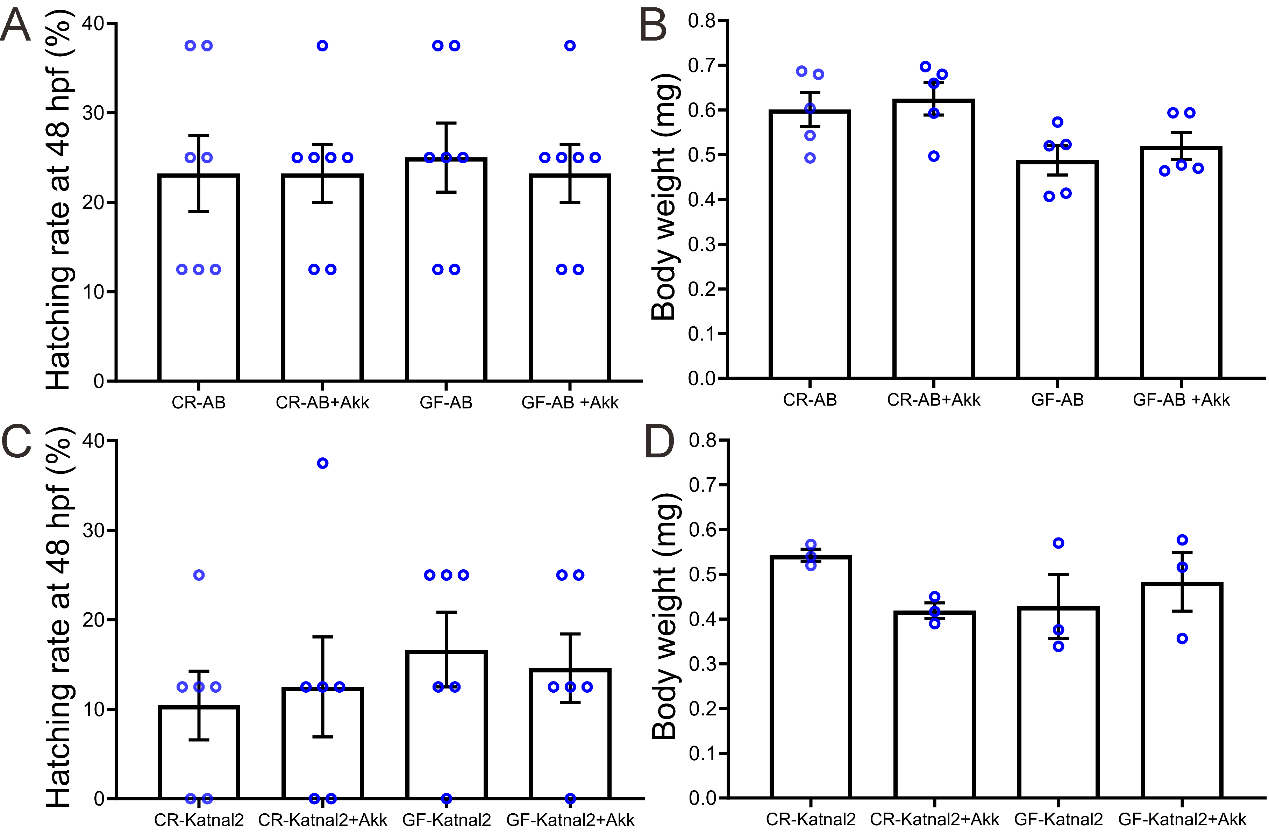


**Figure S2. Developmental indices of AB and *Katnal2* fish after Akk treatment.** (A-B) Hatching rate and body weight of AB groups. (C-D) Hatching rate and body weight of *Katnal2* fish.


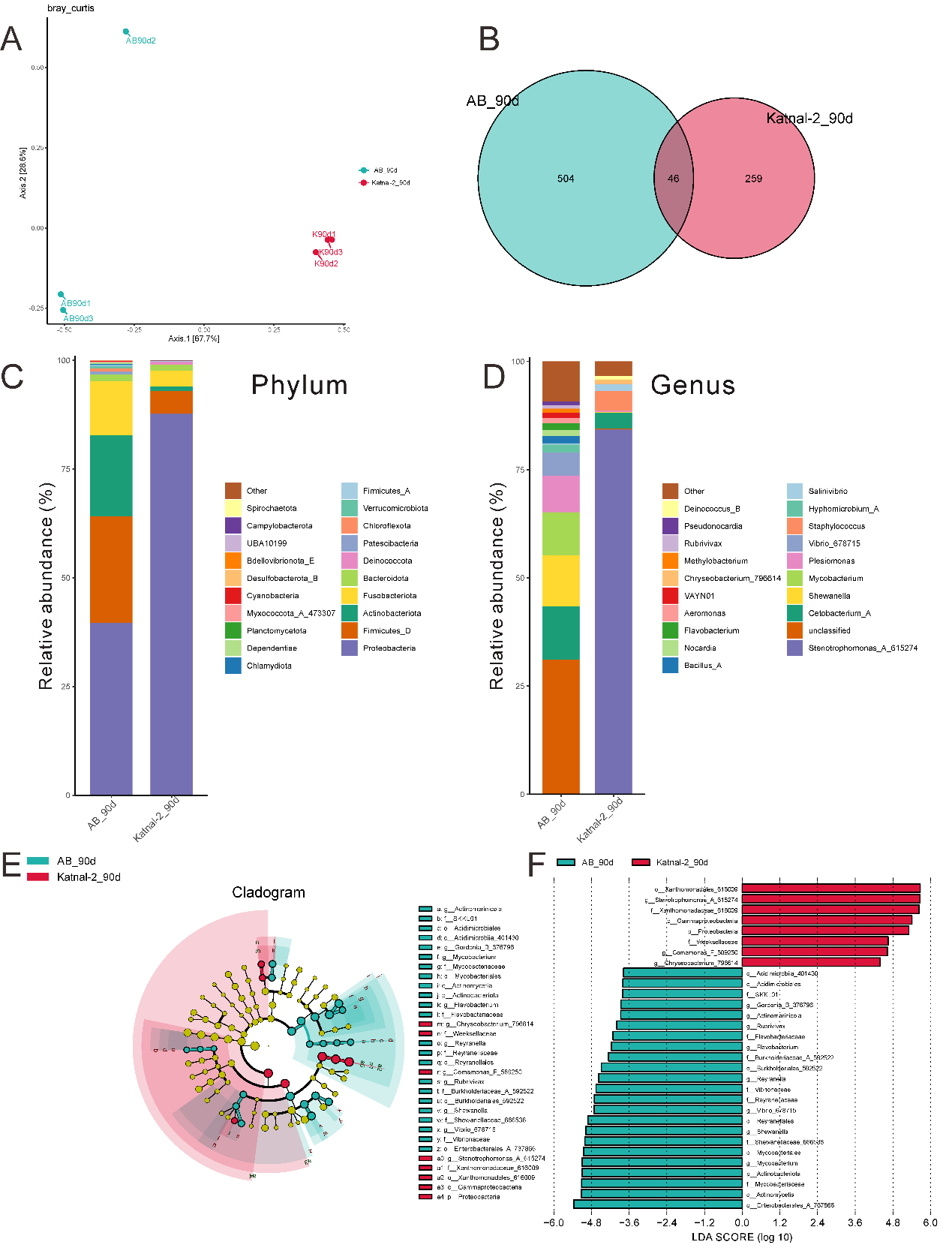


**Figure S3. Gut microbiota composition in adult AB and Katnal2 zebrafish.** (A) PCoA. (B) Venn diagram. (C, D) Phylum- and genus-level composition (n = 3). (E) LEfSe cladogram. (F) LDA histogram (LDA > 2.0).


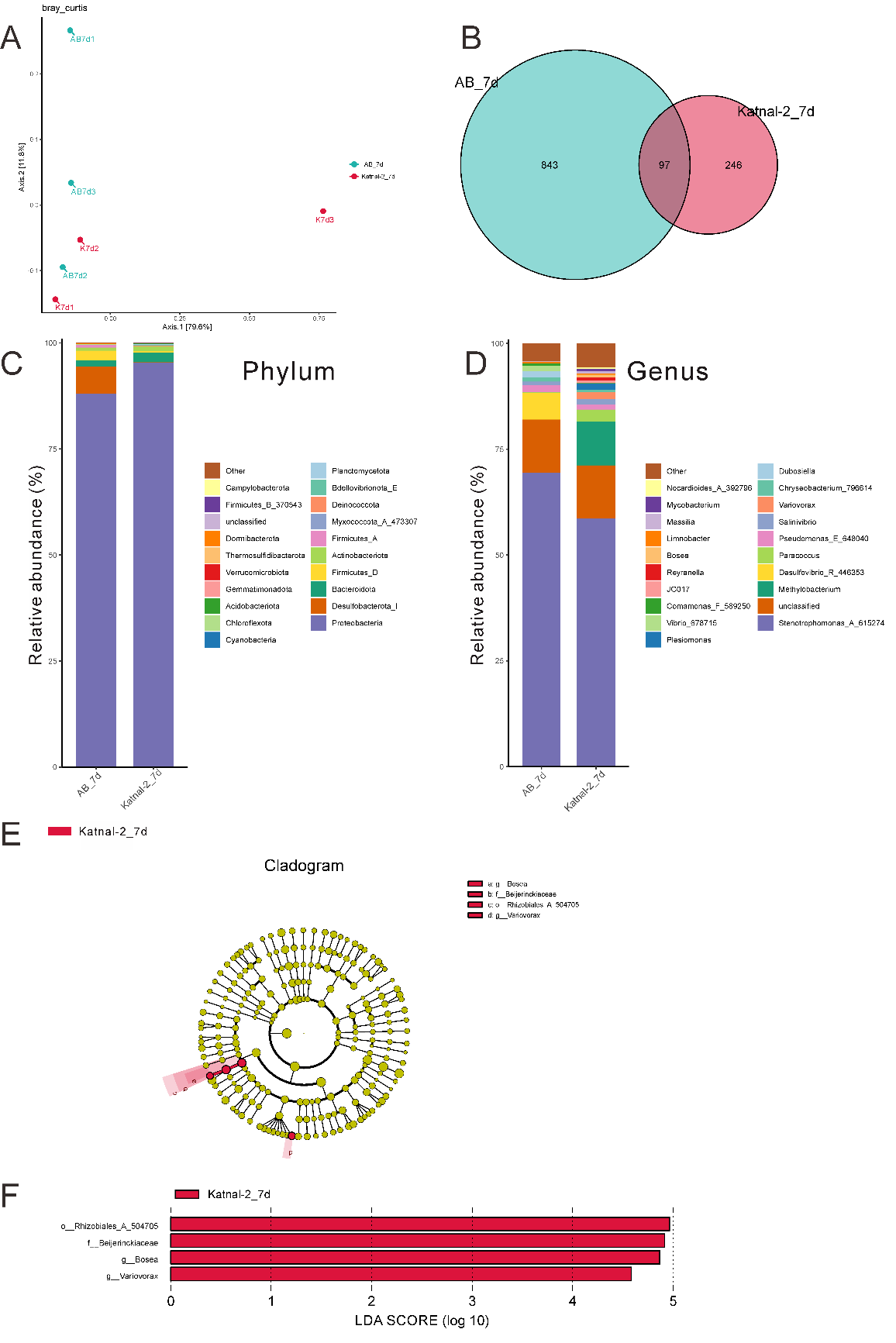


**Figure S4. Composition of microbiota in AB and Katnal2 zebrafish larvae.** (A) PCoA analysis. (B) Venn diagram. (C, D) Gut microbiota composition at phylum and genus levels (n = 3). (E) LEfSe cladogram. (F) Histogram of LDA value distribution (LDA > 2.0).


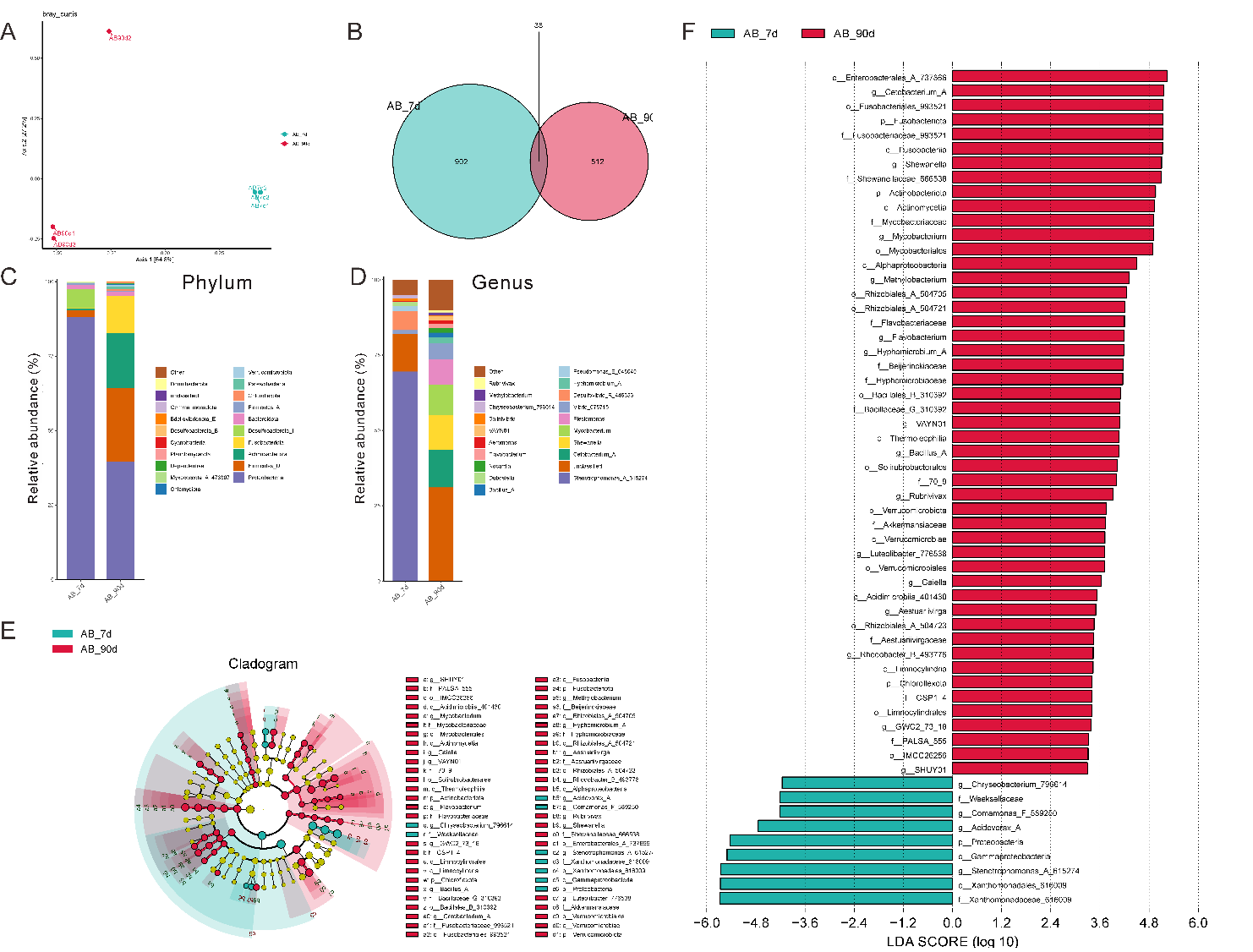


**Figure S5. Distinct microbial communities in larval and adult AB zebrafish.** **(A)**PCoA illustrates the beta-diversity and significant separation of gut microbiota structures between larval and adult stages. **(B)** Venn diagram quantifying the number of unique and shared features between the two developmental groups. **(C, D)**Stacked bar charts depicting the relative abundance and compositional shifts of the dominant bacterial taxa at the (i) phylum and (ii) genus levels across developmental stages (n=3 biological replicates). **(E)**LEfSe analysis identifying the phylogenetically distinct microbial biomarkers that are statistically enriched in each group, visualized *via* a cladogram.**(F)**Histogram of LDA effect sizes, displaying the specific taxa with an LDA score >2.0 that drive the observed compositional differences.


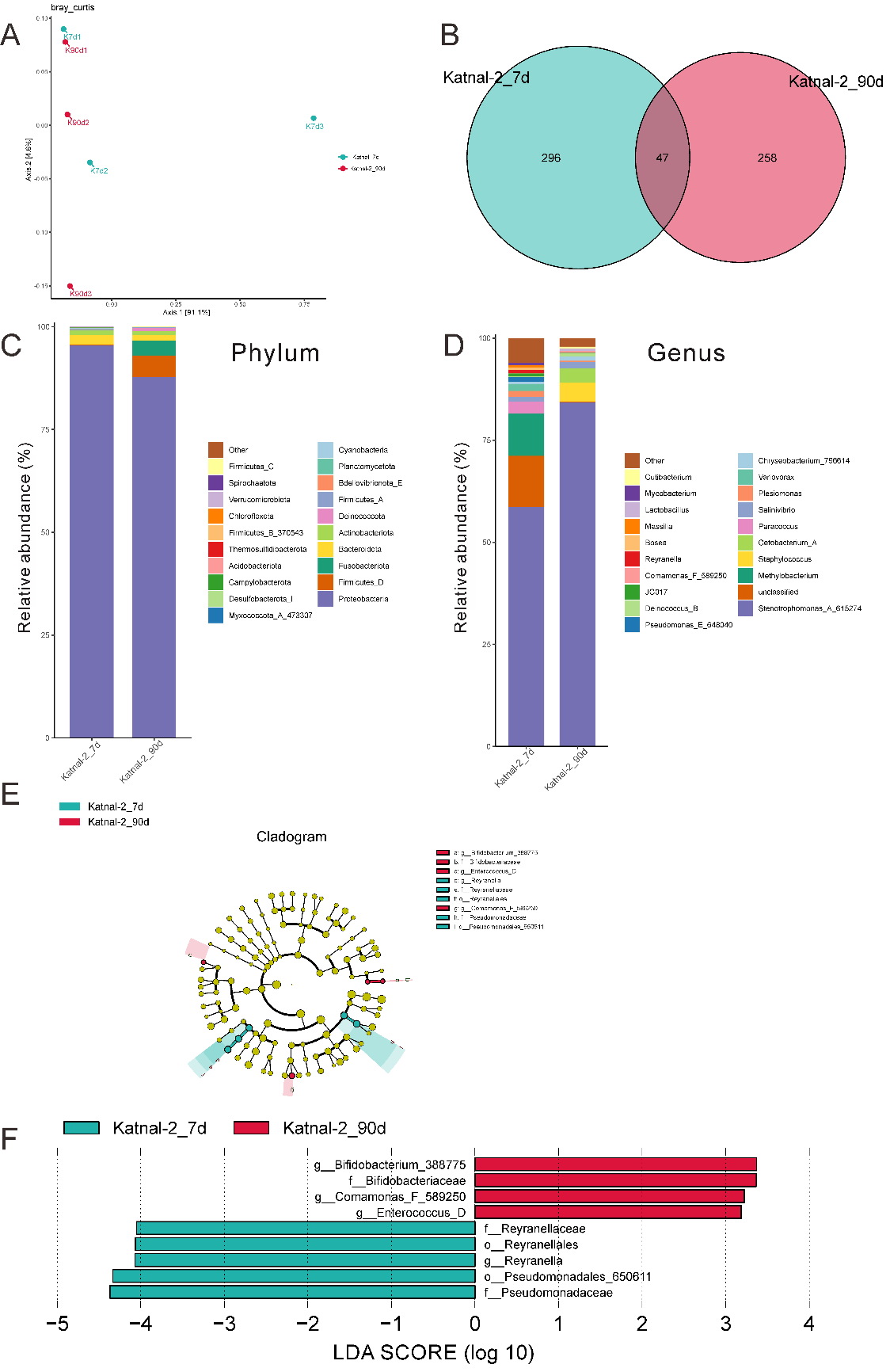


**Figure S6. Composition of microbiota in larval and adult *Katnal2* zebrafish.** (A). PCoA analysis. (B). Venn diagram. (C, D). The accumulation of gut microbiota in zebrafish at the phylum and genus levels (n=3). (E). LEfSe visualization circle diagram. (F). Histogram of LDA value distribution (LDA>2.0).


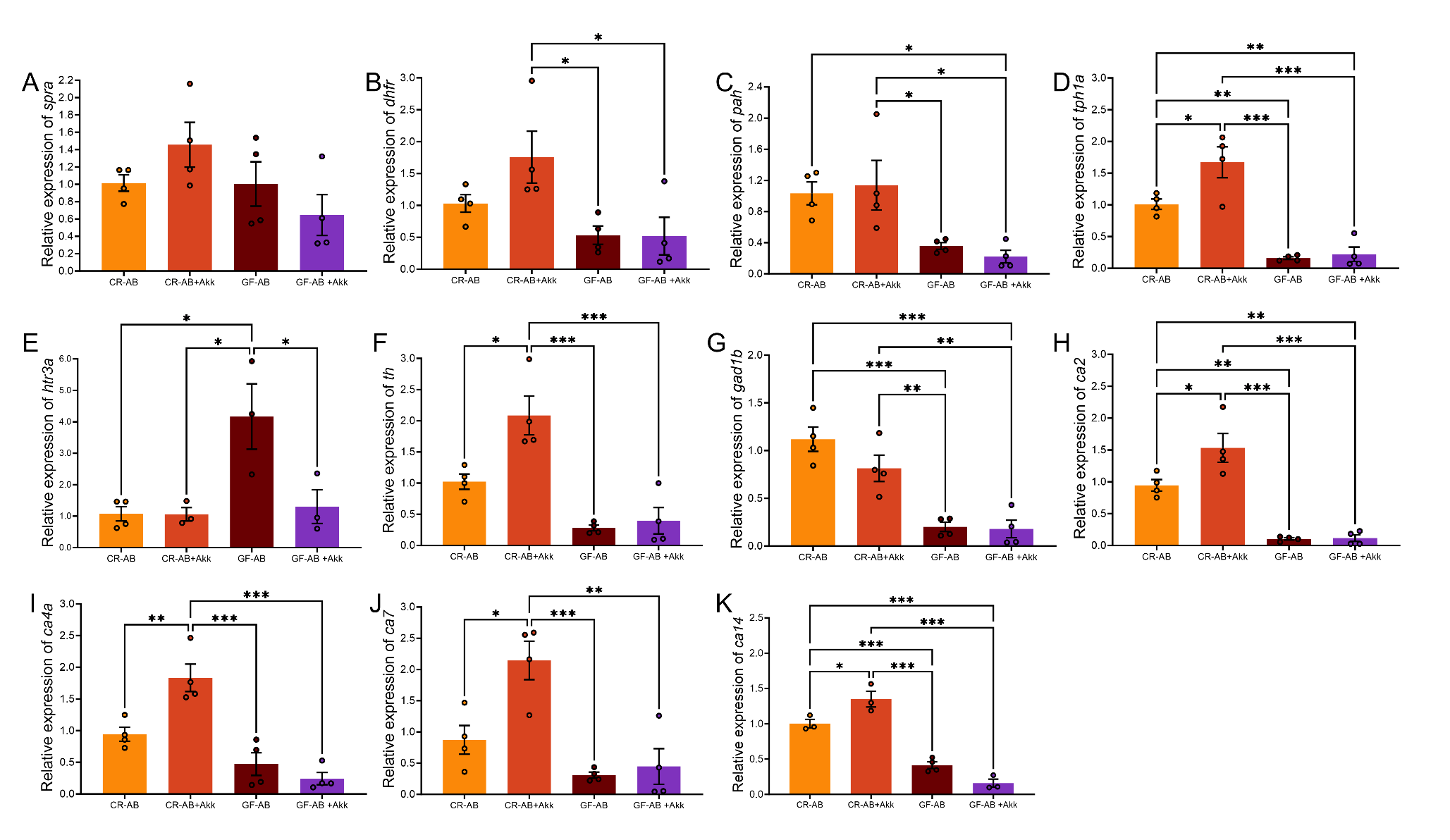


**Figure S7. Expression levels of key genes in AB zebrafish after *Akk* treatment.** Brain development genes: *spra* (A), *dhfr* (B), *pah* (C); neurotransmitter genes: *tph1a* (D), *htr3a* (E), *th* (F); GABA-related *gad1b* (G); carbonic anhydrases *ca2* (H), *ca7* (I), *ca4a* (J), *ca14* (K). There were n=30 zebrafish/replicate sample, and triple samples per group, and significances were **P*< 0.05, ***P < 0.01*, ****P < 0.001* vs. CR-AB, CR-AB+Akk, GF-AB, GF-AB+Akk.


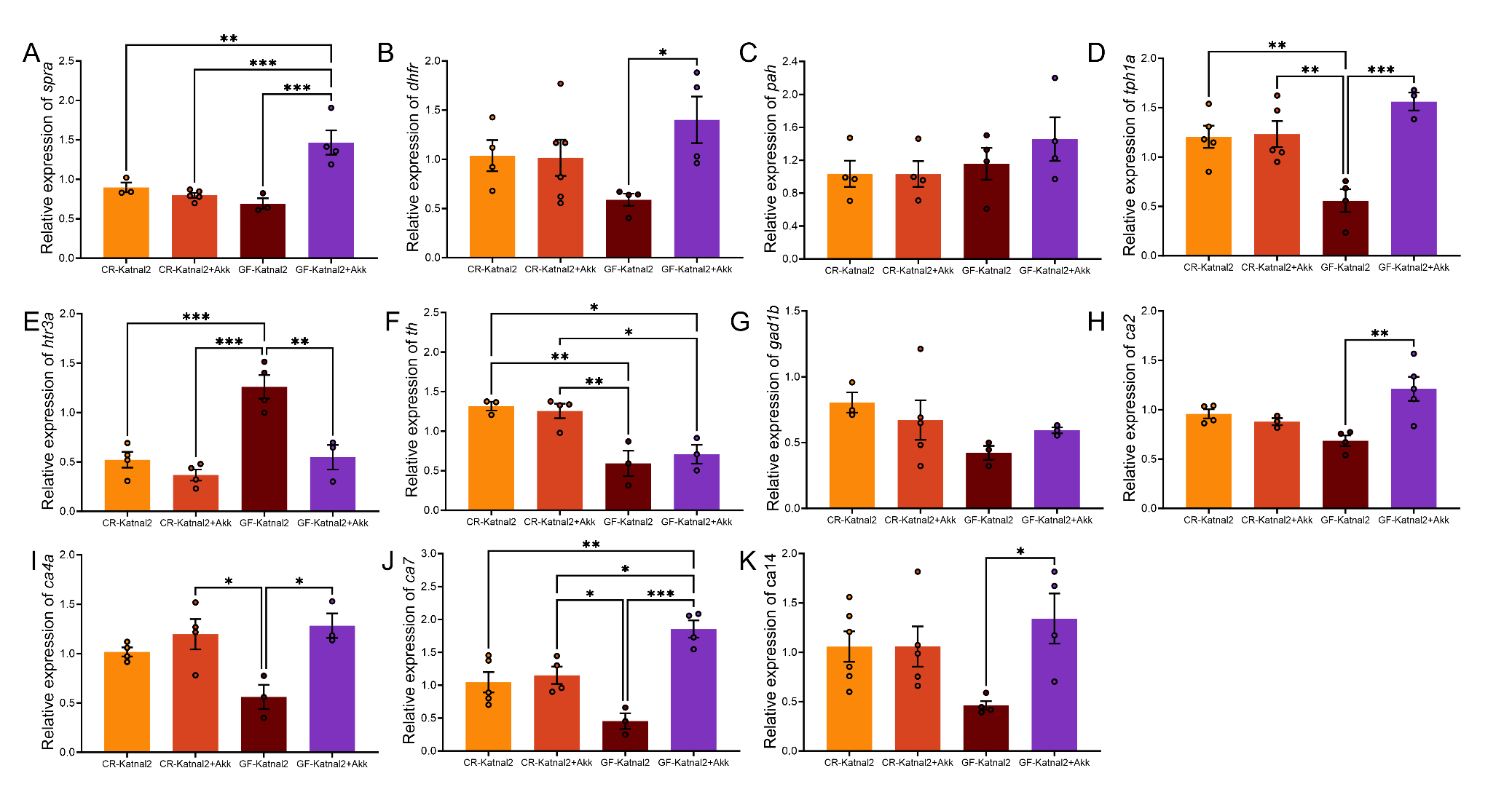


**Figure S8. Expression levels of key genes in *Katnal2* zebrafish after *Akk* treatment.** Brain development genes: *spra* (A), *dhfr* (B), *pah* (C); neurotransmitter genes: *tph1a* (D), *htr3a* (E), *th* (F); GABA-related *gad1b* (G); carbonic anhydrases *ca2* (H), *ca7* (I), *ca4a* (J), *ca14* (K). There were n=30 zebrafish/replicate sample, and triple samples per group, and significances were **P* < 0.05, ***P< 0.01*, ****P < 0.001* vs. CR-Katnal2, CR-Katnal2+Akk, GF-Katnal2, GF-Katnal2+Akk.
